# Retrotransposons: How the continuous evolutionary front shapes plant genomes for response to heat stress

**DOI:** 10.3389/fpls.2022.1064847

**Published:** 2022-12-09

**Authors:** Pradeep K. Papolu, Muthusamy Ramakrishnan, Sileesh Mullasseri, Ruslan Kalendar, Qiang Wei, Long−Hai Zou, Zishan Ahmad, Kunnummal Kurungara Vinod, Ping Yang, Mingbing Zhou

**Affiliations:** ^1^ State Key Laboratory of Subtropical Silviculture, Bamboo Industry Institute, Zhejiang A&F University, Hangzhou, Zhejiang, China; ^2^ Co-Innovation Center for Sustainable Forestry in Southern China, Bamboo Research Institute, Key Laboratory of National Forestry and Grassland Administration on Subtropical Forest Biodiversity Conservation, College of Biology and the Environment, Nanjing Forestry University, Nanjing, Jiangsu, China; ^3^ Department of Zoology, St. Albert’s College (Autonomous), Kochi, Kerala, India; ^4^ Helsinki Institute of Life Science HiLIFE, Biocenter 3, University of Helsinki, Helsinki, Finland; ^5^ National Laboratory Astana, Nazarbayev University, Astana, Kazakhstan; ^6^ Division of Genetics, ICAR - Indian Agricultural Research Institute, New Delhi, India; ^7^ Zhejiang Provincial Collaborative Innovation Center for Bamboo Resources and High-Efficiency Utilization, Zhejiang A&F University, Hangzhou, Zhejiang, China

**Keywords:** transposable element, retrotransposons, LTR, genetic diversity, siRNAs, RdDM pathways, Ty1/copia, Ty3/gypsy

## Abstract

Long terminal repeat retrotransposons (LTR retrotransposons) are the most abundant group of mobile genetic elements in eukaryotic genomes and are essential in organizing genomic architecture and phenotypic variations. The diverse families of retrotransposons are related to retroviruses. As retrotransposable elements are dispersed and ubiquitous, their “copy-out and paste-in” life cycle of replicative transposition leads to new genome insertions without the excision of the original element. The overall structure of retrotransposons and the domains responsible for the various phases of their replication is highly conserved in all eukaryotes. The two major superfamilies of LTR retrotransposons, Ty1/*Copia* and Ty3/*Gypsy*, are distinguished and dispersed across the chromosomes of higher plants. Members of these superfamilies can increase in copy number and are often activated by various biotic and abiotic stresses due to retrotransposition bursts. LTR retrotransposons are important drivers of species diversity and exhibit great variety in structure, size, and mechanisms of transposition, making them important putative actors in genome evolution. Additionally, LTR retrotransposons influence the gene expression patterns of adjacent genes by modulating potential small interfering RNA (siRNA) and RNA-directed DNA methylation (RdDM) pathways. Furthermore, comparative and evolutionary analysis of the most important crop genome sequences and advanced technologies have elucidated the epigenetics and structural and functional modifications driven by LTR retrotransposon during speciation. However, mechanistic insights into LTR retrotransposons remain obscure in plant development due to a lack of advancement in high throughput technologies. In this review, we focus on the key role of LTR retrotransposons response in plants during heat stress, the role of centromeric LTR retrotransposons, and the role of LTR retrotransposon markers in genome expression and evolution.

## Introduction

Eukaryotic genomes contain repetitive elements, such as transposable elements (TEs), that are present in multiple copies throughout the genome. TEs are tandemly arrayed, interspersed throughout the genome, and can be processed as pseudogenes. TEs are major components of eukaryotic genomes and can change their position within genomes (Lisch, 2013; Bourque et al., 2018). TEs were first described in maize by Barbara McClintock in the middle of the twentieth century and she named them jumping genes (Ravindran, 2012; Goodier, 2016). Although TEs are a source of spontaneous mutations, their expression and activity can also increase the stress response to different biotic and abiotic stresses (Ramakrishnan et al., 2021). Moreover, TE specificity has now been associated with the adaptation of plants to a range of these stresses. TEs have deep evolutionary origins and continuous diversification and come in a bewildering variety of forms and shapes (Bourque et al., 2018; Klein and O’Neill, 2018) in most eukaryotic genomes (Wicker et al., 2007; Muñoz-López and García-Pérez, 2010; Gorbunova et al., 2021). TEs are primarily classified into DNA transposons (Class II) and retrotransposons (Class I) based on their mechanism of transposition (Boeke et al., 1985). Both classes are further divided into subclasses based on the mechanism of chromosomal integration. Class I has two major classes, Long Terminal Repeats (LTR) retrotransposons (LTR retrotransposons) and non-LTR retrotransposons (Wessler et al., 1995). LTR retrotransposons and related elements are abundant in plant genomes and include functional genes encoding structural and enzymatic proteins (Galindo-González et al., 2017). LTR retrotransposon mobility is ensured through an RNA intermediate, allowing a copy-and-paste approach for their transposition. Their encoded RNA is reverse transcribed using their own (or not) encoded enzymes that reform a double-stranded DNA from the single-stranded RNA matrix at a new location. LTR retrotransposon integration occurs by cleavage and strand-transfer reaction catalyzed by an integration, similar to retroviruses (Brown et al., 1987). On the other hand, non-LTR retrotransposons include both long interspersed nuclear elements (LINEs) and short interspersed nuclear elements (SINEs)s (Luan et al., 1993).

The two major superfamilies of LTR retrotransposons are Ty1/*Copia* and Ty3/*Gypsy*, which are classified based on the retroviral structural homology and domain order organization of the *pol* gene (Capy, 2005). These LTR retrotransposons exist universally in plant and animal genomes (Malik and Eickbush, 2001; Mangiavacchi et al., 2021). LTR-RTs are more active in plants and their functions are fine-tuned by epigenetic modifications. Although LTR retrotransposons in plants have attracted great attention in recent years, a more comprehensive understanding of the diverse functions of LTR retrotransposons can be gained from further studies. This review provides an overview of the processes associated with LTR retrotransposons involved in precise gene regulation in the plant genome. We also focus on the key role of LTR retrotransposon in plant heat response. Further, we discuss the LTR-derived small interfering RNA (siRNAs), LTR retrotransposon delivery system, centromeric LTR retrotransposons, the application of LTR-based molecular markers, and their contributions towards genome expression and evolution.

## Genome-wide distribution and analysis of LTR families

LTR retrotransposons comprise about 10% to 90% of the total eukaryote genome in most plants. The distribution of LTRs differs among the major families of Ty3/*Gypsy* and Ty1/*Copia* elements in all plant genomes (**Supplementary Table 1**). Ty3/*Gypsy* elements are enriched in euchromatic sub-telomeric regions, whereas, Ty1/*Copia* elements are more frequent in heterochromatic pericentromeric regions (Jedlicka et al., 2019). Moreover, Ty3/*Gypsy* elements play crucial roles in host epigenetic response and are more heterogenous than Ty1/*Copia* elements. Although both families are found in a large number of copies in higher plants, these families were first identified in *Drosophila* (Sant et al., 2000). Members of these superfamilies differ primarily in the arrangement of the gene coding for polymerase function within the polyprotein (POL) region. Ty1/*Copia* elements have a *pol* gene organized as the domains protease (PR), integrase (INT), reverse transcriptase (RT), and ribonuclease H (RNase H) (PR-INT-RT-RNase H). Ty3*/Gypsy* elements are organized as PR-RT-RNase H-INT domains (Sant et al., 2000).

LTR retrotransposons from genomes of about 300 plant species have been identified and are associated with diverse structural, functional annotation, and classification information (Zhou et al., 2021). Thus, this information may provide useful resources for investigating the evolutionary dynamics and functional implications of LTR retrotransposons in plant genomes (Kalendar et al., 2004; Moisy et al., 2014; Kalendar et al., 2020). Moreover, understanding the evolutionary forces governing TE polymorphism is crucial to understanding phenotypic variation in plants (Catlin and Josephs, 2022). Therefore, exploring the role of TEs leading to phenotypic variation and its regulation in plants has significant economic importance in the development of more efficient crops (Kalendar et al., 2008; Malaviya et al., 2021).

## LTRs under heat stress

The impact of TEs on the structure, function and evolution of multiple plant genes have paved the way for epigenetic techniques that address diverse stresses in various crop species. TEs can be highly sensitive to different abiotic and biotic stresses, including salt, cold, heat, wounds, and infections (Mhiri et al., 1997; Ivashuta et al., 2002; Grandbastien et al., 2005; Buchmann et al., 2009; Naito et al., 2009; Ito et al., 2011; Lanciano and Mirouze, 2018). Several studies (**Table 1**) revealed that LTR retrotransposons become activated under certain epigenetic processes, such as siRNA regulation, DNA methylation, LTR retrotransposon integration, and chromatin modification (Grandbastien, 2015; Schorn et al., 2017). Moreover, LTR retrotransposons play a crucial role in the regulation of gene activity at the transcriptional and post-transcriptional level and in genome epigenetic regulation of stress resistance in a wide range of organisms (Galindo-González et al., 2017).

**Table 1:** Summary of LTRs under heat stress and resulting phenotypes.

Target	LTR family	Host plant	Promoter	Findings	Temp point	Reference
*Copia*78	Ty1/*Copia*	*Arabidopsis*	35S promoter	Epigenetic regulation at ambient temperature was transcriptionally activated upon exposure of *Arabidopsis* plants to prolonged heat stress	37°C for 30 h	(Pecinka et al., 2010)
*Copia*-type retrotransposons	Ty1/*Copia*	*Arabidopsis*	35S promoter	Heterochromatin-associated silencing in *Arabidopsis* plants subjected to a particular temperature regime is released in a genome-wide manner	37°C for 15 h	(Tittel-Elmer et al., 2010)
*ONSEN*	Ty1/*Copia*	*Arabidopsis*	*ONSEN* promoter	*ONSEN* insertions confer heat responsiveness to nearby genes	37°C for 24 h	(Ito et al., 2011)
Retrotransposon-like sequences (*LEA, P5CS2*, *AbaH*)	Ty3/*Gyps*y and Ty1/*Copia*	*Pinus sylvestris*	LTR promoters	The transcriptional activation of different types of retrotransposon elements in the Scots pine genome was observed in response to heat-stress conditions	40°C for 16 h	(Voronova et al., 2011)
*ONSEN*	Ty1/*Copia*	*Arabidopsis*	*ONSEN* promoter	Under stress, high accumulation of the transcripts and amplified DNA copies of *ONSEN* were detected in callus	37°C for 24 h	(Matsunaga et al., 2012)
*FaRE*1	Ty1/*Copia*	*Fragaria ananassa*	*FaRE*1 promoter	The promoter of FaRE1 may act as different signal transduction pathways in response to stress	47°C for 32 h	(He et al., 2012)
*ONSEN*	Ty1/*Copia*	*Arabidopsis*	*ONSEN* promoter	Heat-induced transcriptional activation of *ONSEN* family in several species of Brassicaceae	37°C for 24 h	(Ito et al., 2013)
*ONSEN*	Ty1/*Copia*	*Arabidopsis*	*ONSEN* promoter	Plant heat shock transcription factor in periods of heat stress exploits the heat stress response to achieve transposon activation	37°C for 30 h	(Cavrak et al., 2014)
*PtIGF7, PtGypsyX1, PtCopiaX1*	Ty3/*Gypsy* and Ty1/*Copia*	*Pinus sylvestris*	LTR promoters	Stress conditions induced transcriptional activation of a wide range of retrotransposon sequences	40°C for 16 h	(Voronova et al., 2014)
*GBRE-1*	Ty1/Copia	*Gossypium barbadense* and *G. hirsutum*	GBRE-1 promoter	The expression level was increased under the heat-stress condition in *G. hirsutum.*	37°C for 24 h	(Cao et al., 2015)
*ONSEN*	Ty1/*Copia*	*Arabidopsis*	*ONSEN* promoter	Transcriptional activation of *ONSEN* was regulated by a small interfering RNA (siRNA)-related pathway, and the activation may also be induced by stress	37°C for 24 h	(Matsunaga et al., 2015)
*ONSEN*	Ty1/*Copia*	*Arabidopsis*	*ONSEN* promoter	Transposons activated by environmental stress may alter the genome in a potentially powerful manner	37°C for 24 h	(Ito et al., 2016)
*ONSEN*	Ty1/*Copia*	*Arabidopsis*	*ONSEN* promoter	Several *ONSEN* copies in Col-0 were activated by heat stress and maintained their transpositional activity in the progeny	37°C for 24 h	(Masuda et al., 2016)
*ONSEN* (*Copia*78)	Ty1/*Copia*	*Arabidopsis*	*ONSEN* promoter	*ONSEN* heat-responsive elements (HREs) accumulated mutations and lost heat-responsiveness	37°C for 24 h	(Pietzenuk et al., 2016)
*ONSEN*	Ty1/*Copia*	*Brassicaceae*	*ONSEN* promoter	Several new insertions were detected in a regenerated plant derived from heat-stressed tissues and its self-fertilized progeny	37°C for 24 h	(Masuta et al., 2017)
*Copia78* or *ONSEN*	Ty1/*Copia*	*Arabidopsis*	*ONSEN* promoter	Chromosomally integrated LTR retrotransposons consisting of pairwise recombination products were produced in a process comparable to the sexual exchange of genetic information	37°C for 24 h	(Sanchez et al., 2017)
*ONSEN*	Ty1/*Copia*	*Arabidopsis*	*ONSEN* promoter	High inter-and intraplant variation in the number and chromosomal position of new insertions	37°C for 24 h	(Gaubert et al., 2017)
*ONSEN*	Ty1/*Copia*	*Vigna angularis*	*ONSEN* promoter	*ONSEN* element can be fully activated in the calli	40°C for 24 h	(Masuta et al., 2018)
*HuTy1P4*	Ty3/*Gypsy* and Ty1/*Copia*	*Hylocereus undatus*	*Pitaya* LTRs promoter	The Ty1/*Copia* and Ty3/*Gypsy* retrotransposons were usually silent but maybe expressed after exposure to abiotic stresses	45°C for 24 h	(Nie et al., 2019)
*HUO*	Ty1/*Copia*	*Oryza* genus	LTR promoters	Multiple HUO copies may trigger genomic instability through altering genome-wide DNA methylation and small RNA (sRNA) biogenesis and changing global gene expression, resulting in decreased disease resistance and yield	45°C for 10 h	(Peng et al., 2019)
LTRs (*CJHS018732* and *CJHS031206*)	Ty3/*Gypsy*	*Cryptomeria japonica*	LTR promoters	The expression of Ty3/*Gypsy* type retrotransposons was dramatically induced under stress	45°C for 120 min	(Ujino-Ihara, 2020)
*ONSEN*	Ty1/*Copia*	*Arabidopsis*	*ONSEN* promoter	Extrachromosomal DNA of *ONSEN* accumulated in heat-treated plants	40°C for 24 h and 28°C for 24	(Boonjing et al., 2020)
Heat-induced LTRs	Ty3/*Gypsy* and Ty1/*Copia*	*Arabidopsis*	LTR promoters	Heat activation of TEs exhibited a high correlation with the reduction of chromosomal interactions involving peri centromeres	37°C for 72 h	(Sun et al., 2020)
*ONSEN*	Ty1/*Copia*	*Arabidopsis*	*ONSEN* promoter	Under heat stress, loss-of-function of chromomethylase3 (CMT3) mutation led to increased CHH methylation at *ONSEN*	37°C for 24 h	(Nozawa et al., 2021)
LTR/*Copia* and LTR/*Gypsy*	Ty3/*Gypsy* and Ty1/*Copia*	*Arabidopsis*	*ONSEN* promoter	HistoneH1 repressed *Copia* elements by maintaining DNA methylation under heat	37°C for 36 h	(Liu et al., 2021)
*MAGO1/2*	Ty3/*Gypsy*	*Zea mays*	pCsVMV promoter	Argonaute-dependent, RNA-guided mechanism is critical in maize plants to sustain male fertility under stress conditions	38°C for 8 h	(Lee et al., 2021)
*ONSEN*	Ty1/*Copia*	*Arabidopsis*	*ONSEN* promoter	*ONSEN* transcript level was increased in the drd1 mutant relative to wild type under heat stress	37°C for 24 h	(Takehira et al., 2021)

The transcriptional gene silencing of several LTR retrotransposons of *Arabidopsis* is accomplished by the loss of nucleosome and heterochromatin decondensation, which was restored upon recovery from heat stress (Pecinka et al., 2010). This indicates the role of environmental stress leading to epigenetic regulation. Moreover, heat-activated LTR retrotransposons play a crucial role in shaping a genome over an evolutionary period (Wessler, 1996; Masuta et al., 2018). Recently, we reported that the role of two LTR retrotransposons, *PHRE1* and *PHRE2* (Ty3*/Gypsy*), in Moso bamboo (*Phyllostachys edulis*) indicated that the 5’ LTR acts as a promoter and can increase transposition activity during heat stress (Papolu et al., 2021).

A heat-responsive *ONSEN* retrotransposon is conserved among the *Brassica* species, and Adzuki bean exhibited upregulated transcript levels, and full-length extrachromosomal DNA accumulated in the stress-treated plants (Boonjing et al., 2020). The *ONSEN* family in most species of *Brassicaceae* showed integration into active chromatin, which was promoted by heat stress (Ito et al., 2013). Furthermore, there is a correlation between the heat-responsive elements (HREs) of *Copia* families and putative high-affinity heat shock factor binding HREs within the LTRs in seven *Brassicaceae* species. Moreover, the strong HRE of *ONSEN* is conserved over millions of years (Pietzenuk et al., 2016).

The active full-length Ty1/*Copia*, GBRE-1, showed increased expression under heat stress in *Gossypium hirsutum*, and its expression was similar to that of the *ONSEN* retrotransposon (Cao et al., 2015). The heat stress response and heat accumulation of Ty3/*Gypsy* retrotransposon in *Cryptomeria japonica* exhibited differential expression due to preheating treatment with heat shock factors, indicating the impact of LTR retrotransposons in the regulation of heat response systems in plants (Ujino-Ihara, 2020).

Several studies revealed the active role of *ONSEN* in regulating heat stress (Cavrak et al., 2014; Nozawa et al., 2021), including the regulatory role of siRNA. In *Arabidopsis*, *ONSEN* is activated by protracted exposure to heat stress (Ito et al., 2011; Matsunaga et al., 2012; Matsunaga et al., 2015; Ito et al., 2016). The genetic consequences of transposition bursts of the *Arabidopsis* LTR retrotransposon *Copia*78 family generated a novel progeny of chromosomally integrated LTRs consisting of a high frequency of intrafamily recombination and significant sequence diversity of LTR retrotransposons under heat stress (Sanchez et al., 2017). However, the role of LTRs, especially the Ty1/*Copia* and the Ty3/*Gypsy* superfamilies, requires further investigations to reveal their role in heat stress regulation. Such investigations will further the possibilities of developing crops to increase resistance to heat stresses due to global warming.

## The function of LTR-derived siRNA biogenesis

Small noncoding RNAs (sRNAs) are the sequence-specific modulators of gene expression and precisely involved in the regulation of plant immunity (Borges and Martienssen, 2015). sRNAs interfere with the expression of particular genes with complementary nucleotide sequences by degrading mRNA after transcription, thus preventing translation (Laganà et al., 2015). Based on differences in biogenesis and function, sRNAs can be classified into several major classes, including: microRNAs (miRNAs), hairpin-derived siRNAs (hp-siRNAs), natural antisense siRNAs (natsiRNAs), heterochromatic siRNAs (hetsiRNAs) and secondary siRNAs. miRNAs and siRNAs are the two major classes of plant sRNAs. The role of miRNAs in plant development, immunity, and intracellular immune receptors is well documented (Song et al., 2019; Wang S. et al., 2021; Dong et al., 2022). siRNAs are best known for their role in silencing viral RNAs, replication, and genome reprogramming (Kong et al., 2022).

siRNAs are specifically generated from double-stranded RNA (dsRNA) precursors derived from noncoding transcripts, inverted repeat sequences, sense and anti-sense transcripts, and exogenous RNAs (Xie et al., 2004). The dsRNAs are primarily processed into mature 21-24-nt siRNAs by various Dicer-like enzymes (DCL 1-4) and loaded into AGOs to form RISCs. DCL1 processes primary miRNAs into 21-nt-long mature miRNAs. DCL2 is involved in antiviral strategies and cleaves viral dsRNA into 21-22 nt long siRNAs, which target viral transcripts. DCL3 is involved in silencing processes targeting TEs and produces siRNAs approximately 24 nt in length. Finally, DCL4 generates 21-nt transacting siRNAs (tasiRNAs), which silence specific genes. siRNAs can be divided into two main classes: RDR6-dependent secondary siRNAs and RNA polymerase IV-dependent siRNAs (P4-siRNAs) (Kong et al., 2022). Secondary siRNAs are generated by transcripts from noncoding genes, e.g., tasiRNA loci, and protein-coding genes within large gene families, e.g., the nucleotide-binding leucine-rich repeats (NB -LLRs) (Sanan-Mishra et al., 2021). P4-siRNAs, especially 24-nt long, are mainly produced by heterochromatic regions, and TEs are linked to RdDM to induce transcriptional gene silencing (Ito, 2012; Lopez-Gomollon and Baulcombe, 2022).

**Figure 1:**
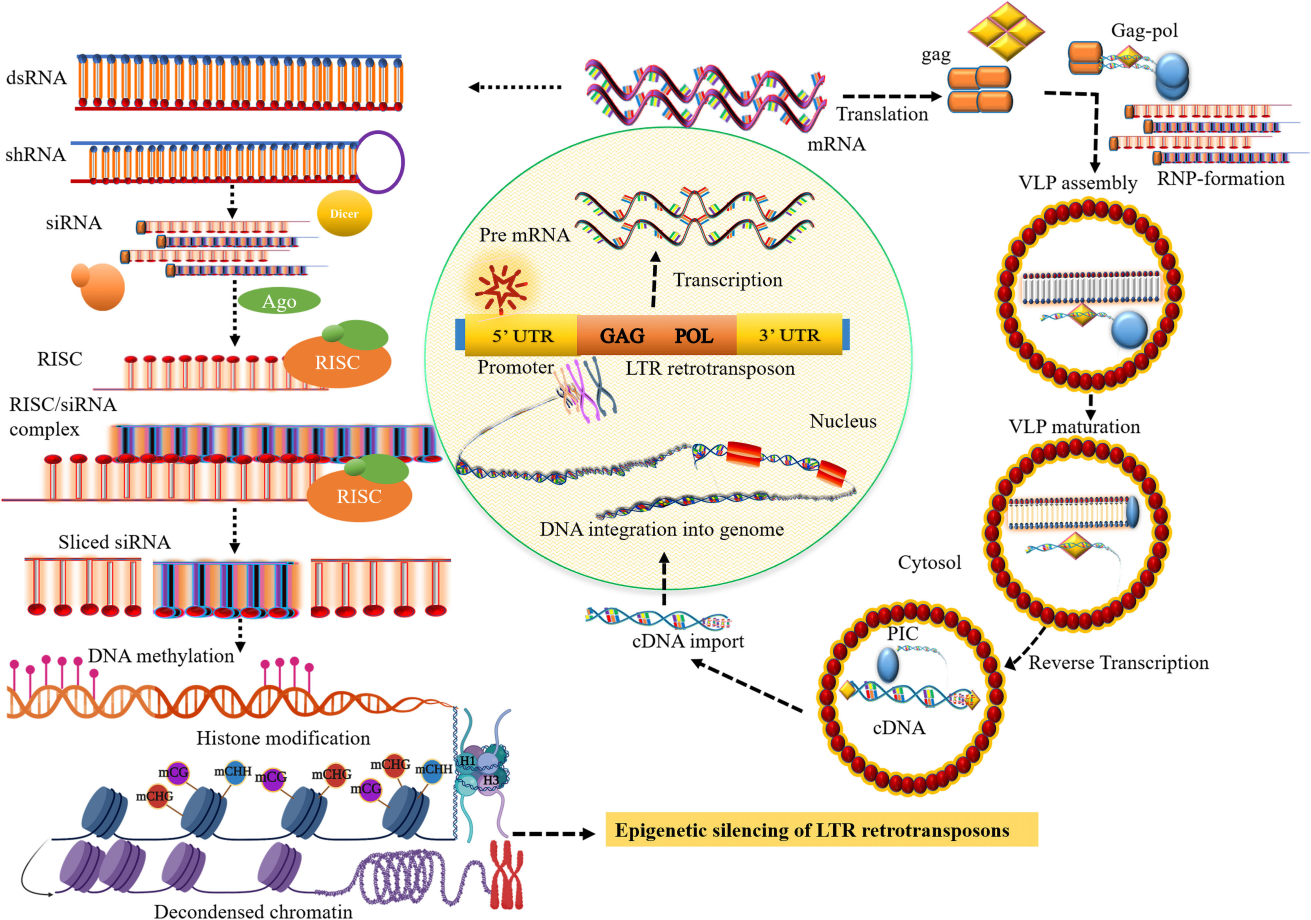
Small RNA biosynthesis and the transposition mechanism of long terminal repeat (LTR) retrotransposons (LTR retrotransposons). The transposition cycle initiates with the transcription of LTR retrotransposon that produces RNA template strand and major structural proteins for reverse transcription. After cleavage of the major structural gag-pol polyprotein by the viral protease (PR) activity, the gag protein-containing capsid and nucleic acid-binding domain involve the formation of cytoplasmic virus-like particles (VLPs). The polyprotein, pol comprising catalytic domain for replication encodes pepsin-like aspartate proteases, integrase, reverse transcriptase, and ribonuclease H proteins, which are crucial for reverse transcription and transposition of retrotransposons. Collectively with the RNA template, reverse transcription most likely takes place within VLPs that produce the cDNA which is then imported into the nucleus. The integrase that involves the formation of DNA nicks at the target sites is inserted into a new chromosomal locus to generate a new copy of retrotransposons and their insertion into the genome. The core siRNA silencing pathway: dsRNA or shRNA is processed into siRNA duplexes by Dicer RNase III. Subsequently, the siRNA or RNA-induced silencing complex (RISC) then binds to the complementary sequence of the target mRNA resulting in the degradation of the target transcript, establishing methylation of DNA through RdDM, and inducing histone modification, and heterochromatin formation.

siRNA pathways are significantly involved in retrotransposon silencing and may mediate different forms of epigenetic regulation in plants (**Figure 1**) (Lippman et al., 2003). In addition, siRNAs derived from TEs act as a trigger for host silencing mechanisms (**Table 2**). For example, siRNA silencing of a different class of LTR-retrotransposon mutants was shown to impact retrotransposon methylation, chromatin remodeling, and histone modification in *Arabidopsis* (Lippman et al., 2003). The mutagenic activity of LTR retrotransposons, especially in the pollen vegetative nucleus of *Arabidopsis*, is suppressed by siRNA silencing that may transmit the TEs to next-generation offspring (Slotkin et al., 2009). Remarkably, siRNAs suppress transposons by RNA-directed DNA methylation (RdDM), thus in turn leading to TEs becoming epigenetically silenced (Nosaka et al., 2012). In maize, loss of RNA-dependent RNA polymerase 2 (RDR2) function in the mediator of paramutation1 (mop1) results in the reactivation of transcriptionally silenced mutator retrotransposon and a substantial reduction in the accumulation of siRNAs. This suggests that the RDR2 pathway is an independent mechanism for silencing LTR retrotransposons in complex genomes like maize (Jia et al., 2019).

**Table 2:** List of small interfering RNA (siRNA), micro RNAs (miRNAs), and small RNAs (sRNAs) derived from LTR retrotransposons and their functions.

Plant species(siRNA/miRNA, size)	Expression pattern	Response	Reference
*Arabidopsis* (siRNAs, 25)	Down	LTR-siRNAs tend to be susceptible to different forms of epigenetic regulation	(Lippman et al., 2003)
*Arabidopsis* (siRNAs, 24)	Up	siRNA produced from TEs activated in the pollen vegetative nucleus can target silencing in gametes	(Slotkin et al., 2009)
Maize (mop1) (siRNAs, 24)	Down	RDR2 pathway is an independent mechanism for silencing retrotransposons, genes, and siRNAs	(Jia et al., 2009)
*Arabidopsis* (siRNAs, 24)	Down	RNA-directed DNA methylation (RdDM) silencing is lower in *Arabidopsis*, which may lead to differential transposon proliferation among species	(Hollister et al., 2011)
MuDR element of maize (siRNAs, 24)	Up	RNA silencing pathway is associated with reduced expression of a regulator of trans-acting siRNA (tasiRNA) pathway and changes in epigenetic regulation of a maize transposon	(Li et al., 2010)
*Arabidopsis* MOM1 (siRNAs, 21-24)	Up	Functional cooperation of MOM1 and Pol-V regulates the degree of transcriptional gene silencing (TGS).	(Yokthongwattana et al., 2010)
*Arabidopsis* (siRNAs, 24)	Up	AGO9 preferentially binds to 24-nt sRNAs and may be a significant source of silencing LTRs in ovule	(Durán-Figueroa and Vielle-Calzada, 2010)
*Arabidopsis ONSEN* (siRNAs, 21-24)	Down	Plays a crucial role in the siRNA pathway in restricting a burst of retrotransposition that may generate novel, stress-responsive regulatory gene networks	(Ito et al., 2011)
Veju element of Wheat (siRNAs, 24)	Down	Intergeneric hybridization and allopolyploidization result in the deregulation of sRNAs and the associated reduction in transposon methylation	(Kenan-Eichler et al., 2011)
Rice (miR820, 24)	Down	The sRNAs silencing might act as a regulator of interactions between hosts and their parasitic elements	(Nosaka et al., 2012)
*Arabidopsis* (siRNA854, 21-22)	UP	Stress response mediated by siRNA854 incorporation into Argonaute1 protein regulates UB1b gene expression during cellular stress	(McCue et al., 2012)
*Arabidopsis* (siRNAs, 21-24)	Up	Nerd protein triggers chromatin-based RNA silencing pathway in plants	(Pontier et al., 2012)
*Arabidopsis* (siRNAs, 21-24)	Up	Distinct functions of Pol IV-RdDM and RDR6-RdDM collectively reestablish transposon methylation and epigenetic silencing	(Nuthikattu et al., 2013)
*Arabidopsis* Evade (EVD) (siRNAs, 21-24)	Up	Potent trans silencing by 24-nt LTR-derived siRNAs can establish functional *de novo* TE silencing at EVD-proximal genes	(Marí-Ordóñez et al., 2013)
Rice (*OsDCL3a*) (siRNAs, 24)	Down	*OsDCL3a*-dependent 24-nt siRNAs derived from transposons influence the expression of nearby genes and affect functional agricultural traits in rice	(Wei et al., 2014)
*Arabidopsis* (easiRNAs, 21)	Down	miRNA-directed 21-nt easiRNA biogenesis preferentially targets long-term heterochromatic silencing and host defense	(Creasey et al., 2014)
*Arabidopsis* (siRNAs, 21-22)	Up	21-22 nt siRNAs are directly incorporated into the AGO6 protein and guide AGO6 to its chromatin targets to establish TE-RdDM function	(McCue et al., 2015)
*Arabidopsis* virus-derived sRNAs (21-24)	Up	Virus-derived 24-nt sRNAs can reinforce VIGS-RdDM as a tool for epigenetic silencing	(Bond and Baulcombe, 2015)
Rice siR815 (21)	Down	TE-siR815-induced suppression of promoter elements of st1 results in WRKY45-mediated disease resistance by RdDM	(Zhang et al., 2016)
*Arabidopsis* sRNAs (23-24)	Up	RDR6-RdDM preferentially targets LTRs and suppressing mobilization in plants is epigenetically inherited in new generations	(Panda et al., 2016)
Strawberry (*fve–miR1511*, 24)	Up	miRNA targets LTR silencing and specifically contributes to genome stability, size, and architecture	(Šurbanovski et al., 2016)
*Arabidopsis* (siRNAs, 24)	Down	siRNAs independent of DCLs (sidRNAs) are associated with Ago4 and may drive heterochromatin DNA methylation	(Ye et al., 2016)
*Arabidopsis* (sRNAs, 24)	Down	Pol IV-dependent sRNAs (P4 RNAs) produced by Pol IV and RDRs may function as trigger RNAs to initiate DNA methylation by dicer-independent RdDM	(Yang et al., 2016)
Moso bamboo (siRNAs,21-24)	Down	Both 21-nt siRNA and-nt siRNAs derived from LTRs may be involved in the epigenetic regulation of host genes and may be responsible for diverse phenotypes	(Zhou et al., 2017b)
*Arabidopsis* (siRNA854, 24)	Up	Transposon-derived siRNA854 produced in the vegetative cell of pollen controls translation of UBP1b connected to triploid seed viability	(Wang et al., 2018)
Sweet pepper (miRNAs, 24 and siRNAs, 24)	Up and down	Differentially expressed 24-nt hetsiRNAs and 21-nt and 24-nt phasiRNAs may be employed to improve the quality and quantity of fruit	(Taller et al., 2018a)
Norway spruce pollen (sRNAs, 24)	Up	Tissue-specific transposon-derived 24-nt sRNAs may provide insights into the functional diversification of sRNAs in TE between gymnosperms and angiosperms	(Nakamura et al., 2019)
Tomato Rider (siRNAs, 24)	Up	Rider stress-induced retrotransposon may be a potential source of epigenetic variations involving siRNAs and RdDM pathway	(Benoit et al., 2019)
*Arabidopsis* embryonic (siRNAs, 24)	Up and down	Chromatin-mediated *de novo* production of sRNAs may provide cell-autonomous homeostasis to help reestablish euchromatic and heterochromatic states	(Papareddy et al., 2020)
*Arabidopsis* (sRNAs, 21-24)	Up	Pol IV switches to generating 21-22 nt siRNAs that are associated with AGO1 and may function in regulating gene expression	(Panda et al., 2020)
*Arabidopsis* (sRNAs, 21-22)	Down	The 21-22nt easiRNAs that depend on RDR6 may be responsible for LTR silencing at transcriptional gene silencing (TGS) and post-transcriptional gene silencing (PTGS) levels	(Lee et al., 2020b)

In *Arabidopsis*, siRNA targeted LTR retrotransposons are associated with reduced gene expression due to RdDM silencing. However, the effect of RdDM silencing was lower in *A. lyrata*, and thus showed differential transposon proliferation among species (Hollister et al., 2011). In addition, the transcriptionally active LTR retrotransposons in *Arabidopsis* produced RdDM-dependent siRNAs, indicating the function of RNA-dependent RNA Polymerase 6 (RDR6) and RNA Polymerase IV (Pol IV). These are independent in the silencing of TEs, in which Pol IV-RdDM functions to initiate TE silencing in an RNA Polymerase II expression-independent manner. In contrast, RDR6-RdDM functions to recognize active Polymerase II-derived TE mRNA transcripts to reestablish DNA methylation and TE silencing (Nuthikattu et al., 2013). Moreover, the targeting specificity of RDR6-RdDM function for full-length LTR retrotransposons in *Arabidopsis* have full-length transposon mRNA to be cleaved by primary 21-22-nt siRNAs and thus the RNA cleavage specificity drives the initiation of epigenetic transcriptional silencing targeted to LTR retrotransposons and transgenes (Panda et al., 2016). The function of DNA methylation to transcriptionally active LTR retrotransposons has demonstrated that mRNA-derived 21-22-nt siRNAs are directly incorporated into the ARGONAUTE 6 (AGO6) protein and in turn guide the AGO6 to its chromatin targets to establish epigenetic transcriptional silencing of TEs in RdDM (McCue et al., 2015).

Recently, Nerd, a plant-specific GW repeat protein triggered by siRNA-dependent DNA methylation in *Arabidopsis*, was found to play a central role in integrating chromatin-based RNA silencing supported by binding both histone H3 and Ago2 proteins and to contribute to siRNA accumulation at a Nerd-targeted locus of LTR retrotransposons. This suggests that RdDM might preferentially target LTR retrotransposons and other repeat sequences (Pontier et al., 2012). The establishment of virus-induced gene silencing (VIGS) mediated RdDM function in *Arabidopsis* requires RNA Polymerase V (Pol V) and *de novo* methyltransferase 2 (DRM2). However, dicer-like-3 and Pol IV pathway components are not required for such functions. Perhaps the DNA methylation in VIGS is guided by virus-derived 21-22-nt siRNAs, thus suggesting VIGS-RdDM is a tool for retrotransposon silencing in *Arabidopsis* (Bond and Baulcombe, 2015). Later, the retrotransposon virus-like particles in *Arabidopsis* are activated by DDM1 mutations, giving rise to 21-22-nt siRNA through RNA-dependent RNA polymerase 6 (RDR6). This suggests that virus-like particle (VLP) DNA could also provide a powerful tool for identifying active LTR retrotransposons from the complex genome and their control at the transcriptional and post-transcriptional levels (Lee et al., 2020a). However, TE-derived siR815 drives RdDM of ST1 promoter and leads to transcriptional suppression of ST1, which abolished the WRKY45 transcription factor in rice resistance to *Xanthomonas oryzae* (Zhang et al., 2016).

The stress-induced full-length *Rider* LTR retrotransposons in the tomato genome indicate that RdDM controls *Rider* activity through siRNA production and DNA methylation, which may contribute to phenotypic variation through epigenetic alteration induced during environmental stress (Benoit et al., 2019). Furthermore, *Arabidopsis* mutations in the Argonaute9 protein (AGO9) indicated that AG09 can interact with 24-nt small RNAs (sRNA) corresponding to LTR retrotransposons expression in the ovule. AGO9 is also necessary for silencing repetitive genomic regions involved in heterochromatin formation. Thus, the AGO9-dependent pathway may be responsible for the epigenetic control of gametogenesis in plants (Durán-Figueroa and Vielle-Calzada, 2010). In a recent report on pepper, pepper-specific heterochromatin-associated 24-nt siRNAs (hetsiRNAs) and 21-24-nt phased siRNAs (phasiRNAs) produced from transposons were preferentially expressed in seeds and placenta, indicating that pepper fruit quality and quantity is associated with changes in sRNA abundance (Taller et al., 2018b). The dynamics of TE-derived embryonic siRNAs in *Arabidopsis* could promote re-methylation of euchromatic and heterochromatic TEs in a new generation, therefore the decondensed chromatin-mediated 24-nt siRNA transcription may provide cell-autonomous silencing of transposons (Papareddy et al., 2020). The TE-siRNAs generated by plant-specific Pol IV can participate in RdDM, whereas other siRNAs and microRNAs (21-22-nt) are associated with *Argonaute1* (AGO1), suggesting that Pol I-dependent 21-22-nt siRNAs may participate in post-transcriptional regulation (Panda et al., 2016; Panda et al., 2020).

In maize, a link between the vegetative phase and the initiation of epigenetic silencing of MuDR retrotransposon is associated with a reduction of mutant expression during plant development. This is associated with an increase in trans-acting siRNA (*LRR*s) levels, which in turn is responsible for silencing epigenetic regulation of the MuDR element (Li et al., 2010). The regulatory interplay between *MOM1* mutants of LTR-retrotransposon in *Arabidopsis* and RNA polymerase-V may regulate the intensity and siRNA accumulation at the transgenic locus and the transcriptional gene silencing at the locus is accompanied by DNA methylation (Yokthongwattana et al., 2010). The heat-induced *ONSEN* retrotransposon in *Arabidopsis* showed its accumulation was stimulated in mutants deficient in the biogenesis of siRNAs, suggesting a considerable role of the siRNA pathway triggered by environmental stress during retrotransposition (Ito et al., 2011).

In wheat, high-throughput sRNA sequencing in parental, hybrid, and allopolyploid plants showed that miRNAs and the TE-derived siRNAs respond differently to changes at the ploidy level, and the siRNA pools were significantly reduced upon allopolyploidization. This, in turn, causes siRNA deregulation and the associated reduction in CpG methylation of LTR retrotransposons, which may contribute to genome instability at the initial stage of speciation (Kenan-Eichler et al., 2011). The Fatima family LTR retrotransposons of polyploid wheat are highly specific to B-genome and proliferated before allopolyploid wheat formation (Salina et al., 2011). Likewise, in hexaploidy wheat TriRe-1, LTR retrotransposons have a specific amplification history of B-genome progenitors, implying that genome-specific TriRe-1 may be utilized for the development of wheat molecular markers (Monden et al., 2014b).

In rice, a transposon produces microRNA820 (miR820) to suppress host silencing. The miR820 negatively regulates the expression of *de novo* DNA methyltransferase gene OsDRM2, indicating that transposon-derived siRNA silencing might act as a regulator of interactions between the host and their TEs (Nosaka et al., 2012). The Dicer-like 3 homolog OsDCL3a produces 24-nt siRNAs that target gibberellin (GA) and brassinosteroid (BR) homeostasis-related genes by association with TEs, which suppress the expression of nearby genes and may control important agricultural traits in rice (Wei et al., 2014). Whereas Dicer-like (DCL) proteins and 24-nt siRNAs are not required for DNA methylation at RdDM target loci, P4 sRNA transcripts generated by Pol IV and RNA-dependent RNA polymerases (RDRs) may function as RNA-triggered gene silencing of retrotransposons to initiate DNA methylation through the RdDM pathway (Yang et al., 2016). However, the biogenesis of TE heterochromatin-associated siRNAs in *Arabidopsis* is mechanically distinct from gene-regulating microRNAs (miRNA) or tasiRNAs. This suggests that the TE-derived siRNA854 regulates UBP1b mutant gene expression during the stress response, and the accumulation of siRNA854 is under the same trans-generational epigenetic regulation and inheritance pattern as the *Arabidopsis* LTR retrotransposons (McCue et al., 2012). Evd LTR-derived 24-nt siRNAs can silence transactive Evd copies in *Arabidopsis*. Reciprocal crossing between F11 and F14 plants resulted in the silencing of all F11-derived Evd copies. In addition, an Evd RNA and 3′ gag-derived siRNAs of 21-22 nt were below detection in F1 plants, indicating effective trans silencing by LTR-triggered 24-nt siRNAs (Marí-Ordóñez et al., 2013).

In *Arabidopsis*, Post-transcriptional gene silencing (PTGS) mediated by miRNA-directed siRNA biogenesis specifically targets retrotransposon transcripts, whereas transcriptional gene silencing (TGS) of LTR retrotransposons is mediated by 24-nt heterochromatic (het) siRNA. Together, LTR retrotransposons give rise to the most abundant 21-nt epigenetically activated siRNAs (easiRNAs) in *ddm1* and methyltransferase1 (*met1*) mutants, and in the nucleus of pollen grains and callus cultures. Consequently, this supports an antagonistic relationship between PTGS and TGS in plants (Creasey et al., 2014).

In moso bamboo, both 21-nt siRNA and 24-nt siRNA have targets within LTR regions of retrotransposons. The high number of siRNAs derived from LTR retrotransposons may be responsible for diverse phenotypes of moso bamboo (Zhou et al., 2017a). The silencing mechanism of LTR retrotransposons is mediated by the most abundantly expressed miRNA, fve-miR1511. This fve-miR1511 is generated from a single locus that specifically targets LTR transcripts at the PBS site for methionyl initiator tRNA, which is essential for reverse transcription. This may contribute to features such as genome stability size and architecture in strawberries (Šurbanovski et al., 2016). The distinct class of 24-nt siRNAs independent of Dicer-like 3 (DCL3) is associated with effector AGO4 and is capable of driving DNA methylation and is subsequently subjected to 3’-5’ exonucleolytic activity for maturation. Therefore, this class may be the initial trigger of *de novo* DNA methylation (Ye et al., 2016).

In addition, the transposon-associated sRNAs in pollen and cell culture of Norway spruce are responsible for tissue-specific and environmentally induced gene repression. This may provide insights into the diversification process of sRNA in transposon silencing between angiosperms and gymnosperms (Nakamura et al., 2019). The enhanced retrotransposon expression in *Botrytis cinerea* leads to the suppression of plant defense-related genes during infection. Retrotransposons are pathogenicity factors that manipulate host gene expression by encoding trans-species sRNAs (BcsRNAs) and therefore have a broad impact on host-microbe interactions and pathology (Porquier et al., 2021).

Previously, understanding of the sRNA activity in plants generally came from their prominent functions in plant development. Now, there is a greater understanding of the complex molecular mechanisms involved in sRNA biogenesis and function in plants. sRNAs play a significant role in the diversification and specialization of gene silencing. This is because there are several pathways for sRNA biogenesis and function, which are related to evolution. However, most sRNA classes contribute to biotic and abiotic stress and transgenerational inheritance, and the stability of acquired sRNA-based responses has not been characterized.

However, unless sRNAs in isolated cell types and single cells can be profiled, understanding of the specificities and interplay between the different gene-silencing mechanisms operating in plant cells will remain limited. Therefore, focused research on the aspects described above is necessary to manage stress-induced agricultural losses and the development of stress-resistant crops.

### Nanoparticle-based LTR retrotransposon delivery system into plants

**Figure 2:**
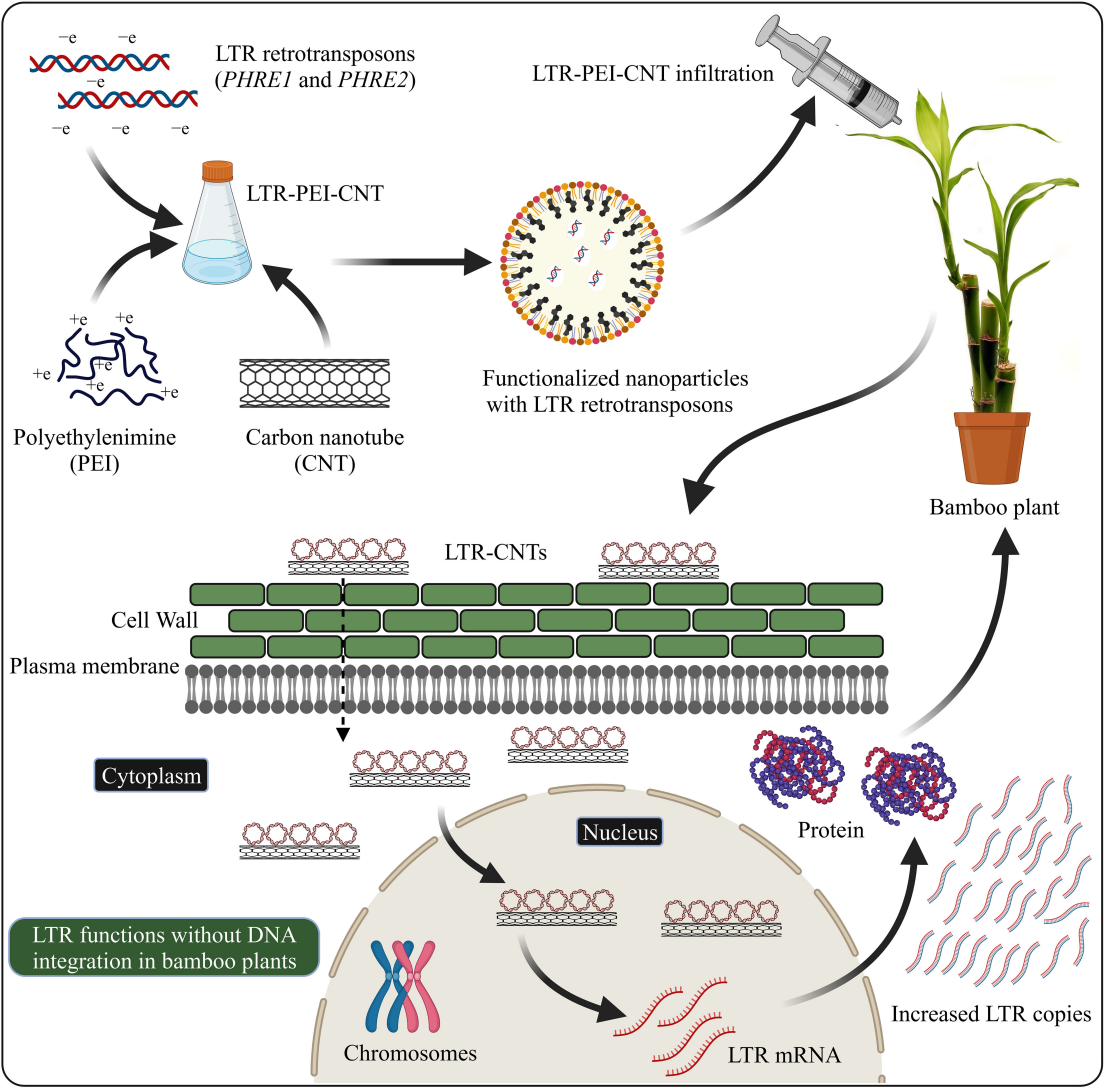
LTR retrotransposons with carboxylated carbon nanotubes (COOH-CNTs) and polyethylenimine (PEI) delivery into bamboo leaves. Covalently modified COOH-CNTs with PEI carrying a net positive charge are incubated with negatively charged LTR vectors. Bamboo leaves infiltrated with LTR–CNTs produce LTR transcripts and proteins and increased LTR copies, without the genome integration. The schematic representation is based on Demirer et al., (2019a) and Papolu et al. (2021) and created with BioRender.com.

Nanomaterial-mediated delivery of biomolecules and therapeutics has been extensively studied in animals, but its potential for plant-based systems lags behind (Demirer et al., 2019a). Several previous studies have used nanoparticles to deliver plasmid DNAs (Cunningham et al., 2018; Wang J. W. et al., 2019; Lv et al., 2020), proteins (Wang J. W. et al., 2019; Wang J. W. et al., 2021), small interfering RNAs (Demirer et al., 2020), and intact plant cells (Serag et al., 2011; Demirer et al., 2019a). Carbon nanotubes have been used to perform stable genetic transformation in bacterial (Castillo et al., 2021; Weise et al., 2022) and mammalian (Golestanipour et al., 2018) cells. In our recent study, we used for the first time an efficient polyethylenimine (PEI)-walled carbon nanotube (SWNT) diffusion method to introduce the LTR retrotransposon plasmid DNA into Moso bamboo plants without transgene integration (Papolu et al., 2021) (**Figure 2**). We found that internalization of nanoparticles in the intact plant cells resulted in increased GFP expression in the leaves after 72 hours. The carbon nanotubes enable the transport of plasmids without integration of transgenes into crop plants (Kwak et al., 2019). GFP were expressed in various tissues such as roots, leaves, protoplasts, and immature tissues (Ali et al., 2022). Enhanced GFP expression in leaf protoplasts by the use of carbon nanomaterials has been demonstrated in arugula (*Eruca sativa*), *Gossypium hirsutum* (cotton), and wheat (*Triticum aestivum*) (Demirer et al., 2019b; Kwak et al., 2019). The use of nanoparticle-mediated transformation has also been demonstrated for siRNA gene silencing production (Demirer et al., 2020; Zhang et al., 2021). Further focused studies on LTR retrotransposon delivery system are required to explore the molecular mechanisms of LTR retrotransposons in the plant genome.

## Roles of centromere-specific retrotransposable elements

LTR retrotransposons are greatly responsible for plant genome evolution and are enriched in the pericentromeric region of host genomes. Active retrotransposable elements are also highly mutagenic and often target protein-coding genes for insertion. In addition, these elements cause chromosome breakage, illegitimate recombination, and genome rearrangement. Therefore, active retrotransposable elements are recognized to play a central role in maintaining chromatin structures, centromeric functions, and regulation of gene expression in their hosts (Shapiro, 2014). Moreover, they are largely responsible for plant genome size variation (Girard and Freeling, 1999; Slotkin and Martienssen, 2007). Centromeric sequences play a central role in chromosome distribution during the mitotic and meiotic cell lifecycle (de Castro Nunes et al., 2018). Centromeric retrotransposons (CR) were first discovered in the grass as centromere-specific sequences (Miller et al., 1998; Presting et al., 1998). Remarkably, in plants, they are usually surrounded and dispersed by LTR retrotransposon sequences (Neumann et al., 2011). The centromere-targeting retrotransposable elements can replace centromeric tandem repeats that bind centromere-specific proteins and may act as a substrate for the efficient repair of frequent double-stranded breaks (Presting, 2018). The centromere-specific histone H3 (CENH3)-associated sequences of centromeric retrotransposons and satellite DNAs are the important structural elements in epigenetic centromere function (Keçeli et al., 2020). Retrotransposable elements can be used to deduce centromere positions, as some elements target active centromeres during integration (Presting, 2018). However, the roles of retrotransposable elements in centromere functions remain unclear. Centromere-targeting elements may be able to replace centromeric tandem repeats. Therefore, centromeric retrotransposons of several plant species have been investigated during the last two decades.

In wheat, the FISH analysis revealed that the sequence of pHind258 was homologous to integrase and the LTRs of centromeric Ty3-*gypsy* retrotransposons of cereal species (Ito et al., 2004). A 67-kb clone (R11H) containing Ty3/*gypsy* retrotransposon-related sequences was also identified, which showed strong hybridization signals on the centromeres (Fukui et al., 2001). The expansion of centromeric retrotransposon sequences on dicentric chromosomes to chromosome arms and the formation of multiple centromeres in wheat-rye hybrids may be responsible for chromosome breakage in the next-generation offspring and may be associated with chromosomal rearrangement, stability, and novel chromosome formations (Guo et al., 2016).

In cotton species, centromere-associated sequences are composed of A and D genomes, and the location of the functional centromere co-localizes with centromere retrotransposon hybridization on metaphase mitotic chromosomes. Additionally, FISH and dot-blot hybridization revealed that centromere retrotransposons are present only in D-genome diploid species, indicating that retrotransposons may have invaded the A-genome centromere from the D genome during allopolyploidization (Luo et al., 2012). In addition, LTRs generated from a sequenced bacterial artificial chromosome (BAC) were located in the D progenitor in *Gossypium raimondii* but not in the A progenitor *G. herbaceum*, indicating that the centromeric regions of triploid cotton may be derived from D progenitor (Zhang et al., 2014). Moreover, centromeric retrotransposable elements from the different progenitor genomes may become activated during genomic instability following allopolyploidization (Divashuk et al., 2016). Thus, allopolyploid offers an opportunity to understand the evolution of centromeric sequences from resident TEs (Hartley and O’Neill, 2019).

In maize, centromeric retrotransposons represent a transcriptionally active component of centromeres from a wide range of angiosperm species and play a central role in plant centromere evolution (Neumann et al., 2011). However, a recent study revealed that the centromeric retrotransposons can give rise to CRM1 and CRM4 tandem repeats in maize. Nevertheless, maize centromeres are fluid genomic regions whose borders are heavily influenced by the interplay of retrotransposons and epigenetic marks. Distinct CRM1TR sequence variation may lead to gene conversion, which is the main cause of sequence variation and may increase the size of the satellite repeat locus (Sharma et al., 2013). Furthermore, FISH and chromatin immunoprecipitation (ChIP) with anti-CENH3 antibodies in maize and soybean revealed that centromeres differ in size and contain a higher density of CENH3 chip reads, indicating that the tandem satellite repeats and interspersed centromeric retrotransposons may be shaped primarily by retrotransposons (Wolfgruber et al., 2009; Tek et al., 2010). Additionally, various repetitive elements in maize, including centromeric retrotransposon, CentC, and CentA, are found preferentially near the centromeres of the A chromosome hybridized to distinct sites from centromere on the B chromosome, revealing a high concentration of centromeric repeats at the major location on the B chromosome (Lamb et al., 2005). A comparative genomic analysis of centromeric retrotransposons in maize revealed that the maize B chromosome co-existed with the A chromosome during retrotransposition, suggesting that the B chromosome had its origins from A chromosome elements (Theuri et al., 2005). The cores of maize centromeres contain primarily CentC arrays and a cluster of centromere-specific retrotransposons of maize. The structural relationship between CentC, centromeric retrotransposons, and CENH3 was visualized by sequential detection procedure on stretched centromeres, demonstrating that the maize centromeres constantly incorporate oat CENH3 nucleosomes (Jin et al., 2005).

Tobacco cell lines have been identified with an expression of a HaloTag7-fused CENH3 centromeric-tandem repetitive DNA sequences located with CENH3 by a HaloTag7-based chromatin affinity purification system. Further, FISH and ChIP analysis indicated that repeats were chromosome-specific centromeric retrotransposons (Nagaki et al., 2012). Moreover, the centromeric retrotransposons derived from BAC clones act as centromeric DNA sequences in tobacco and the estimated amplification timings of centromeric retrotransposons were different in the two ancestral diploid species of tobacco, indicating that retrotransposons accumulate especially in CENH3-binding regions of tobacco species (Nagaki et al., 2011).

In *Brassica* species, centromere retrotransposons are the major repeats in centromeric and pericentromeric heterochromatin, and the distribution of the species in allotetraploid relatives indicates that repetitive elements are A-genome specific (Lim et al., 2007). In addition, ChIP and immunostaining analysis with anti-CENH3 antibodies showed that both centromere-specific retrotransposons and centromeric tandem repeats represent a dominant component of the diploid and allotetraploid *Brassica* species and are directly associated with CENH3 proteins (Wang et al., 2011). Recently, the centromeric-specific retrotransposon in *Brassica* species showed that the centromeric repeats spread and proliferated between the diploid species possessing A, B, or C genomes after polyploidization, implying that centromeric retrotransposons are particularly important in the evolution and polyploidization of the *Brassica* genome (Wang G.-X. et al., 2019). Furthermore, the repetitive elements in Brassica species that are conserved in pericentromeres, sub-telomeres, and telomeres rapidly diverged during the evolution of A/C and B genome lineages. Furthermore, these repeats may be associated with genomic stability and may provide insights into genome evolution during Brassica polyploidization (Koo et al., 2011). BACs derived from the rapid proliferation of nested LTR retrotransposons in *Brassica* species may play an evolutionarily important role in the formation of centromere regions (Wei et al., 2013).

In rice, the contribution of LTR retrotransposons to the evolution of gene structure and function indicates that Ty3/*Gypsy* elements are more abundant than Ty1/*Copia* elements, and the intrachromosomal distribution of retrotransposons across chromosome 10 is non-random with the highest density being present in the pericentromeric region (Gao et al., 2004). Moreover, the structural features of LTR retrotransposons in rice indicated that centromeric retrotransposons and CentO satellite repeats are harbored in the core region of the rice chromosome 4-specific centromere, indicating the fragmental duplication of arrays of satellite repeats is mainly responsible for the amplification of centromere satellite DNA and rapid reshuffling of CentO satellites (Ma and Bennetzen, 2006). Although the centers of rice centromeres are occupied by a CentO satellite repeat and a centromere-specific retrotransposon, the CentO satellite is quantitatively variable among 12 rice chromosomes and is interrupted by centromeric retrotransposons, therefore suggesting that CentO satellite and centromere-specific retrotransposons may be the key DNA components for centromere function in rice (Cheng et al., 2002).

The position of CENH3 nucleosomes in rice centromeres is regularly spaced with 155-bp periodicity on CentO satellite repeats but not on non-CentO sequences, suggesting that centromeric repeats evolve for the stabilization of CENH3 nucleosomes (Zhang et al., 2013). Evidence also suggests that suppression of LTR retrotransposon proliferation through the formation of heterochromatin may be an advantage in large genomes in eukaryotes that have a high content of LTR retrotransposons (Cossu et al., 2017). The centromeric retrotransposons of rice are enriched with heterochromatin and its constitutive sequences are transcribed in all the tested rice organs. The centromeric transcripts are differentially processed into sRNAs, indicating a crucial role in the RNAi-mediated pathway for heterochromatin formation and centromere function (Neumann et al., 2007).

Recently, the phylogenetic relationships of centromeric retrotransposons in grasses show that horizontal transfer of centromeric retrotransposon between oryzoid (rice) and panicoid (maize, sorghum, *Setaria*, *Panicum*, and *Coix*) lineages and interelement recombination are important factors in the evolution of centromeric retrotransposons (Sharma and Presting, 2014).

In sugarcane, the characterization of centromere-associated DNA sequences indicated that centromeric retrotransposable elements and centromeric tandem repeats may directly interact with CENH3 in sugarcane centromeres (Nagaki and Murata, 2005). Moreover, the centromeric satellites had the formation and evolutionary stability for 7 million years and exhibited different ploidy levels and unusually longer monomeric repeats that lacked translation phasing on the CENH3 nucleosomes. This indicates that they originated from a retrotransposon and may form extrachromosomal circular DNAs (eccDNAs) (Huang et al., 2021).

In the grass family, centromere-specific retrotransposons discovered in BAC clones revealed that both centromere-specific and non-centromere-specific repeats are the primary DNA elements of maize centromeres and may play a significant role in grass family evolution (Nagaki et al., 2003). Similarly, a centromeric LTR retrotransposon of *Brachypodium distachyon* derived from centromeric BAC sequences was found in high copy number and is enriched in *B. distachyon* centromeric regions, indicating that *Brachypodium* centromeric retrotransposons are highly divergent among other grass species (Qi et al., 2013).

In the potato genome, retrotransposon-related sequences and satellite repeat-based centromeres can rapidly proliferate from neocentromeres by *de novo* amplification and can associate with the CENH3 nucleosome (Gong et al., 2012). The LTR retrotransposons identified using BAC inserts in Beta species have a chromodomain that is highly similar to centromeric retrotransposons in rice, maize, and barley. Based on sequence diversity, LTRs may have been transposed within the last 60 000 years, indicating that their large-scale genomic organization and transcriptional activity may play an important structural role in centromeres of chromosomes (Weber and Schmidt, 2009).

The annotations and comparison of the centromeric region of *Coffea*, which is rich in several centromeric retrotransposon family elements, showed that the role of LTR retrotransposons may be more diverse in plants and may extend beyond the chromodomains (de Castro Nunes et al., 2018). The centromeric region of tomato chromosome 12 is composed of nested repeat sequences, including LTR retrotransposons and chloroplast DNA insertions. A block of CAA trinucleotide microsatellite repeats was found in the centromere and pericentromeric region of chromosome 12, suggesting that microsatellite arrays like CAA blocks may be a component of tomato centromeres (Yang et al., 2005). A high copy number of tandem repeats in *Allium* species is located in all chromosomes and differs in sequence, structure, chromosome level, and genome organization. These repeats are transcribed and associated with the insertions of retrotransposons and organelle DNA, which can be used for future applications of its association with kinetochore protein CENH3 (Kirov et al., 2020). Likewise, the chromosomal organization of centromeric retrotransposons in the genomes of *Allium cepa* and *A. fistulosum* are localized in centromeric regions and the chromosomes of *A. fistulosum* are expressed less in centromeric regions and were abundant in other chromosomal regions (Kiseleva et al., 2014). Holocentromeres in *Rhynchospora pubera* is composed of centromeric units interspersing the gene containing chromatin. A cell-dependent shuffling of multiple centromeric units results in the formation of functional centromeres during mitosis; genome-wide analysis indicated that different types of holocentromeres may exist in different species, with and without repetitive elements among eukaryotes (Marques et al., 2015).

In *Arabidopsis*, the centromere-enriched retrotransposons are significantly diverged between two different species and can target their integration preferentially into the centromere region on each of the different chromosomes in the karyotype (Birchler and Presting, 2012). Furthermore, the structure and organization of centromere-specific retrotransposons and CentO-F satellites in *Oryza brachyantha* indicate that CentO-F satellites are located within the chromosomal regions and are characterized by tandemly repeated satellite DNA flanked by centromeric retrotransposons. This may explain its potential impact on functional centromeres in *Oryza* species (Yi et al., 2013). FRetro3 centromeric retrotransposons are located in the functional domains of *O. brachyantha* centromeres and have replaced centromeric retrotransposons of rice as dominant centromeric retroelements in *Oryza* species (Gao et al., 2009). The retrotransposon of *A. lyrata* Tal1 was introduced into *Arabidopsis* by tissue culture-mediated transformation and showed that the highest retrotransposed copies were found in centromeric repeats of *Arabidopsis*, which suggests dynamic controls for the evolution of the retrotransposon-rich heterochromatin regions (Tsukahara et al., 2012). Furthermore, the structural heterozygosity and chromosomal rearrangements of tissue-specific retrotransposons and tandem repeat copy number in *Aegilops speltoides* indicate that the tissue-specific pattern of retrotransposons emerges during cell proliferation and this may reflect the reorganization of individual genomes under rapid environmental changes (Shams and Raskina, 2018). However, significant advancements in epigenetics and different types of plant centromeres may be essential to increase the number of sequenced genomes (Oliveira and Torres, 2018).

Over the last two decades, several varied approaches have been used to study the genomes of many plant species. Studies on agriculturally important plant species are particularly important. Following genome sequencing of crop plants, genome sequencing within the genus should be the next targeted research for genomic analysis. Further research should be conducted on genome organization and comparisons at the chromosome, sequence, functional, and evolutionary levels (Voronova et al., 2020).

Several studies demonstrated that LTR retrotransposons participate in centromere-specific transposition and may be a driving force in plant centromere evolution. However, there are many mechanisms involved in the organization of genome functions and in maintaining complex programs of genome organization. Therefore, studies resolving the questions above require novel technologies in molecular biological, cytogenetic, biochemical, and genetic methods. Such studies may provide a clearer understanding of the relationship between plant evolution and LTR retrotransposons.

## Applications of LTR-retrotransposon as molecular marker system

Retrotransposable and related elements are highly abundant in eukaryotic genomes and insert into new genomic locations by a mechanism that involves the reverse transcription of an RNA intermediate. Changes in the copy number of repeat elements and internal rearrangements on both homologous chromosomes occur after the induction of recombinational processes during the meiotic prophase. The insertion of LTR retrotransposons is random and occurs in the transposition process in the continuous evolution of a species. This can provide a wealth of information for the study of evolution, species, and genome differentiation.

Retrotransposon-based DNA marker applications have become a key element of research on genetic variability and diversity (Vuorinen et al., 2018; Ghonaim et al., 2020; Kalendar et al., 2021b). The scope of their usage includes creating genetic maps and the identification of individuals or lines carrying certain genetic polymorphic variations (Khapilina et al., 2021a). LTR retrotransposon-derived molecular genetic marker systems have been employed in deciphering the genetic diversity of crop plants (Kalendar et al., 1999; Kalendar and Schulman, 2006; Kalendar et al., 2011; Kalendar et al., 2018; Kalendar et al., 2021a). The retrotransposon-based marker systems are highly effective in detecting the effects of environmental stress on retrotransposon activation (Kalendar et al., 2008; Belyayev et al., 2010). Moreover, the detection of TE expression, including polymorphisms and the diversity of the transposon transcriptional landscape, may provide new insight into host-TE interactions (Lanciano and Cristofari, 2020). In addition, LTR retrotransposons are associated with key genes involved in potential applications of genome assembly, genome variation, gene tagging, and functional analysis of genes, indicating their crucial role as markers in molecular breeding (Potter, 2005).

In pepper (*C. annuum*), LTR retrotransposons were inserted 6 million years ago and exhibit chromosomal insertional preferences, which may be a useful tool to design species-specific retrotransposon-based markers (Yañez-Santos et al., 2021). The combination of active LTR retrotransposons and Inter-Retrotransposon Amplified Polymorphism (IRAP) markers (Kalendar et al., 1999; Hosid et al., 2012) may be a suitable system for genetic fidelity assessment of tissue-culture-generated plants in sugarcane (Shingote et al., 2019) and better germplasm management in *Xanthosoma* and *Colocasia* (Doungous et al., 2015). The IRAP marker system in LTR retrotransposon insertions of flax genome appeared to be suitable for the identification of retrotransposon polymorphisms and showed a high level of plant adaptation in a radioactive environment (Smýkal et al., 2011; Lancíková and Žiarovská, 2020). The IRAP and REMAP markers of the cassava genome produced high polymorphism and may be suitable for the investigation of genetic diversity and relationships among cassava cultivars (Kuhn et al., 2016). A comparative analysis of two LTR retrotransposons, BARE-1 and Jeli, may provide a potential source of polymorphic Sequence-Specific Amplification Polymorphism (SSAP) markers for genetic diversity in diploid wheat (Konovalov et al., 2010). The LTR retrotransposon based SSAP markers in cashew and myrtle genomes exhibited a significantly higher proportion of polymorphic markers than those of AFLP (Waugh et al., 1997; Syed et al., 2005; Woodrow et al., 2012).

The genetic maps generated with several retrotransposon-based markers such as iPBS (inter-Priming Binding Site) (Kalendar et al., 2010; Doungous et al., 2020; Khapilina et al., 2021b) and REMAP (REtrotransposon-Microsatellite Amplified Polymorphism) (Kalendar et al., 1999) exhibited regions of different marker densities, indicating that the distribution of retrotransposons in lentil is non-random and widespread throughout the lentil genome. This may be useful in lentil breeding by marker-assisted selection (Rey-Banos et al., 2017). The development of Retrotransposon-Based Insertion Polymorphism (RBIP) markers (Flavell et al., 1998) derived from sweet potatoes can determine intraspecific variability. These markers can also be used as core primer pairs for evaluating genetic diversity and constructing linkage maps of various plant species, guiding breeding and germplasm research (Meng et al., 2021). The RBIP marker was shown to be duplicated several times during the development of Asian pear cultivars and may provide a comprehensive picture of the complex relationship and evolution of *Pyrus* species (Jiang et al., 2015). Likewise, genome-wide analysis of RBIP markers in the *Melilotus* genome revealed considerable polymorphism information content (PIC), indicating that these markers are highly informative and may be used for implementing genetic improvement in the *Melilotus* genus (Ouyang et al., 2021). Furthermore, RBIP markers used for DNA profiling of Japanese, Chinese, and European pear cultivars revealed that retrotransposons have transposed during Asian pear evolution or reflect the genetic relationship between Asian and European pears. Thus, suitable combinations of retrotransposon insertions may be useful for cultivar-specific DNA markers (Kim et al., 2012). The polymorphism markers generated from several retrotransposon families and the effectiveness of the dominant (IRAP) and codominant (RBIP) marker systems for assessing the genetic diversity among different potato varieties were compared. Distinct DNA profiles for Ty1/*Copia* and Ty3/*Gypsy* retrotransposons are active in the genome and may contribute to potato genome organization (Sharma and Nandineni, 2014). High-throughput RBIP data analysis indicated that may strongly support the model of independent domestications for *Pisum sativum* species, which in turn provides a broad understanding of the diversity and evolution of *Pisum* (Jing et al., 2010). Likewise, a wide variety of LTR retrotransposon-based markers generated from peas, broad beans, and Norway spruce may be useful in revealing polymorphisms associated with the corresponding retrotransposons within the *Pisum* genus (Pearce et al., 1999). The non-random distribution of abundant LTR retrotransposons within the lentil genome indicates that defective and non-autonomous retrotransposons are highly frequent and maybe a suitable source of genetic markers for further genetic analysis (Rey-Banos et al., 2017). The novel Ty1/*Copia* and Ty3/*Gypsy* LTR retrotransposons derived from *Lilium* species indicate that they were non-autonomous retrotransposons. IRAP analysis using the LTR sequence of these retrotransposons may provide a new approach to analyzing the species relationship among *Lilium* species (Lee et al., 2015). In *Cleistogenes songorica* and strawberry genomes, various LTR retrotransposon-based molecular markers were developed and exhibited a high level of polymorphism frequency and high transferability of polymorphic primer pairs. This suggests that RBIP markers may be useful in future studies on genetic diversity, QTL mapping, population structure, and the evolution of germplasm accessions in *C. songorica* and related grasses (Monden et al., 2014a; Ma et al., 2022). Several LTR retrotransposon markers derived from chokecherry genome sequences indicated that retrotransposon markers in map construction and genetic mapping may facilitate genetic research in Rosaceous species (Liang et al., 2016).

## Role of LTR retrotransposons in plant evolution

Evolution is primarily a change in physiological and genetic composition; therefore, variation is a significant process in evolution. Like in most eukaryotes, TEs are the most variable parts of the plant genome (Lisch, 2013). TEs can make dramatic differences in the overall architecture of the genomes of even closely related plant species. Moreover, TEs make up most of all plant DNA (Bennetzen et al., 2005). Gene inactivation is one of the most common TE-induced phenotypic changes. Therefore, the propensity of some TEs to insert into or near genes has been successfully utilized for generating new null mutations (Hsing et al., 2007; Settles et al., 2007; Candela and Hake, 2008), and this is also a major driver of genome size evolution (Hawkins et al., 2006; Neumann et al., 2006; Piegu et al., 2006) Therefore, the evolutionary potential of TEs, especially LTR retrotransposons, should be thoroughly explored to gain a better understanding on the evolutionary characteristics of plants. Retroviruses and LTR retrotransposons share similar gene architecture, but LTR retrotransposons lack the envelop gene and an extracellular stage in their lifecycle. It has been proposed that these retroviruses emerged from the LTR retrotransposon family Ty3/Gypsy by acquiring the envelope gene (Malik and Eickbush, 2001), but this evolutionary relationship is not confirmed.

Genome relationships and LTR retrotransposon diversity can be used to understand the genomic relationship among the members of a genus or family in plants. Recently, genome relationships and LTR retrotransposon diversity in three cultivated *Capsicum* strains were analyzed and a close relationship among the species was revealed (de Assis et al., 2020). Moreover, genome-wide analysis of LTR retrotransposons and their impact on evolution has been explored in several plants (Roulin et al., 2009; Beulé et al., 2015; Giordani et al., 2016; Mascagni et al., 2017; Keidar et al., 2018; Liu et al., 2018; Akakpo et al., 2020; Mascagni et al., 2020; Ouyang et al., 2021). TE amplification is the main mechanism behind plant genome size increase and evolution (Gantuz et al., 2022). The proliferation of LTR retrotransposons is related to genome reorganization caused by hybridization or polyploidization (Vicient and Casacuberta, 2017). Moreover, allopolyploidization is associated with rapid structural and functional alterations of genomes (Leitch and Leitch, 2008) and this is recognized as the major mechanism behind adaptation and speciation in the plant kingdom (Ramsey and Schemske, 1998). In addition, polyploidy increases genome size and activates TE amplification, and the resultant genome rearrangement may alter their balance in epigenetic silencing (O'Neill et al., 1998; Ozkan et al., 2001; Madlung et al., 2005; Petit et al., 2007; Parisod et al., 2009). TEs are known to associate with recombination-driven sequence loss that leads to major structural changes (Parisod et al., 2010). In plants, TE abundance is correlated with the recombination rate of some TE families (Daron et al., 2014). In maize, LTR retrotransposons are enriched in regions of low recombination (Stitzer et al., 2021). Moreover, a negative correlation between LTR retrotransposons and recombination was also reported in many other plant species, such as soya bean, rice, and bread wheat (Tian et al., 2009; Tian et al., 2012; Daron et al., 2014). Angiosperm genomes are unstable at the level of chromosome number, genome size, and repetitive DNA content; most genes are found as single-gene groups surrounded by nested TEs (Sanmiguel and Bennetzen, 1998). Furthermore, in maize, any two alleles of the same gene diverged >2 million years ago (Wang and Dooner, 2006). Although gene content and organization are mostly similar, variation in copy number and gene order has been observed in grass plants (Bennetzen, 2007; Springer et al., 2009). However, variation in copy number and its influence on genome order and evolution should be explored to gain a better understanding of the influence of LTR retrotransposons in plant evolution.

In general, LTR retrotransposons are one of the key elements that drive evolution by mechanisms of recombination and gene duplications. Moreover, TEs affect the genome when the mobile elements are closer to the genome or even from a considerable distance. This is because TEs can move. Therefore, TEs, especially LTR retrotransposons, have a significant role in the evolution of the plant kingdom because of their wide occurrence. Further focused studies are required to explore the role and the exact process of LTR retrotransposons in plant evolution, which may provide further insight into the molecular mechanisms of evolution in the plant kingdom.

## Future perspective

1. Retrotransposable elements represent up to 90% of the total genome in most plants. Several studies describe the role of LTR retrotransposons in epigenetic regulation. Exploring the role of retrotransposable elements leading to phenotypic variation and its regulation in plants may have significant economic importance in the field of plant breeding and agriculture.
2. Investigations on the role of LTR retrotransposons, especially the Ty1/*Copia* and the Ty3/*Gyps*y superfamilies, may reveal their roles in heat stress regulation, which will provide a further understanding of the possibilities of developing smart crops that are resistant to heat stresses due to global warming.
3. Genetic engineering methods and epigenetic modifications using LTR retrotransposons may have future scope in the field of smart agriculture by developing smarter crops.
4. Further research should focus on profiling sRNAs in isolated cell types and single cells. This may further understand the specificities and interplay between the different gene-silencing mechanisms in plant cells.
5. There is currently a limited understanding of most sRNA classes that contribute to biotic and abiotic stress and the transgenerational inheritance and stability of acquired sRNA-based responses. This should be a focus of further research in the development of stress-resilient crops and plant breeding in general.
6. LTR retrotransposons participate in centromere-specific transposition and may be a driving force in plant centromere evolution. Further studies should focus on genome organization and comparisons at the chromosome, sequence, functional, and evolutionary levels.
7. Genome-sequencing studies on agriculturally important plant species are important; genome sequencing within the genus should be targeted for subsequent research.
8. Significant advancements in epigenetics and different types of plant centromeres are required to increase the number of sequenced genomes. This increase should further understand the relationship between plant evolution and LTR retrotransposons.
9. Further investigations are necessary to gain a better understanding of the variation in copy number of LTR retrotransposons and its influence on evolution and genetic variation.
10. TEs, especially LTR retrotransposons, contribute significantly to intraspecific phenotypic variation in plants. Therefore, understanding the dynamics governing LTR retrotransposons is a crucial research focus for evolutionary biologists.

## Conclusion

Retrotransposons are a class of mobile genetic elements that are universally distributed in plant genomes. Their distribution and transposition activities are significantly associated with plant evolution. Several studies of LTR retrotransposons have provided valuable insights into the mechanism of the genome evolution of plants. The genomes of most plant species exhibit dynamic variations in size and other structural features of LTRs. In chromatin modification, reduced DNA methylation often promotes the expression of retrotransposons. A wide variety of genetic factors are responsible for retrotransposon expressions, such as miRNAs, ncRNAs, piRNAs, RdRPs, risiRNAs, siRNAs, ta-siRNAs, ra-siRNAs, nat-siRNAs, dsRNAs, endo-siRNAs, viRNAs, heterochromatin, DNA methylation, histone post-translational modifications, and gene silencing pathways. Moreover, the potential biological functions of plant sRNAs to acquire information from different tissues and shift it across generations may improve future plant research. The development of RNA biogenesis mechanisms leads to the regulation of biological processes coupled with plant development and environmental responses. Retrotransposable elements, considered a kind of genetic pool, have tremendous potential in genome analysis, biodiversity research, gene mapping, gene cloning, and functional analysis.

A high proportion of LTR retrotransposons are involved in multiple epigenetic mechanisms, including stress tolerance, transpositional activity, regulation of gene expression, DNA methylation, histone modification, and chromatin remodeling and their interconnected networks in the plant genome. Increasing research interest in such epigenetic mechanisms may contribute to a greater understanding of their central role in genome organization and evolution. Therefore, an integrated TE database with epigenetic information will be a valuable resource for future research focused on assessing the possible contribution of LTR retrotransposons to develop single-molecule real-time sequencing and transcriptome variations resulting from advancements of genome annotation and investigations of plant genetic diversity. Moreover, advancement in the forthcoming reference genomes in association with novel sequence technologies may lead to the implementation of long-read sequencing. This will further enhance understanding of various aspects of genome disruption of LTR retrotransposons.

Environmental stresses affecting crops grown under field conditions are a major threat in the global warming era, and the activities of retrotransposons show a close relationship with such stresses. During environmental stress, LTR retrotransposons are more active and induce mutational and insertional polymorphisms. LTR retrotransposon-mediated molecular genetic markers are a highly polymorphic and efficient system. Moreover, as this does not influence genetic structure across species, DNA marker investigations will be a promising tool for exploring crop diversity and germplasm. Furthermore, several studies revealed that centromere-specific retrotransposons are conserved in pericentromeres, sub-telomeres, and telomeres and have rapidly diverged during the evolution of A, B, and C diploid genome lineages. Moreover, recent developments in genomics-based on whole-genome sequencing and 3D nuclear organization, allele-specific histone modification, and RNA Pol II binding profiles may facilitate the understanding of epigenetic regulation of differential gene expression between homologous chromosomes.

Another consideration is nanoparticle-based biomolecule delivery systems. In these systems, biomolecules such as DNA, RNA, and protein can be efficiently delivered and incorporated into the plant genome. This method can be utilized to make desirable epigenetic modifications in crop plants. In addition, high-throughput sequencing technology combined with artificial intelligence approaches for big data analysis may be beneficial in providing a more comprehensive picture of the interplay between LTR retrotransposon-induced epigenetic changes. Further collaborative studies are required to understand the complexity of LTR retrotransposons in evolutionary and organismal biology.

## Author contributions

PKP and MR planned, designed, and wrote the review. PKP, MR, SM, QW, RK, PY, and MZ outlined and edited the review. PKP, MR, RK, QW, SM, LHZ, ZA, KKV, PY, and MZ edited and revised the review.
