# Supplementary material for: Retrotransposons: How the continuous evolutionary front shapes plant genomes for response to heat stress
Source: Front Plant Sci. 2022 Dec 9;13:1064847. doi: 10.3389/fpls.2022.1064847 (PMC9780303; doi:10.3389/fpls.2022.1064847)
Supplement: Supplementary file 1 [file Table_1.docx]

**Supplementary Table 1.** Distribution of LTRs differs among the major families of Ty3/*Gypsy* and Ty1/*Copia* elements in monocots and dicots.

| **Monocots** | | | | | | | | | |
| --- | --- | --- | --- | --- | --- | --- | --- | --- | --- |
| **Common name** | **Scientific name** | **Total**  **genome**  **Size**  **(Gbp)** | **Total TE % in genome** | | **Total % of LTR retrotransposons in genome** | | **Total % of super families in LTR-retrotransposons** | | **Reference** |
|  |  |  | **%** | **Gbp** | **%** | **Gbp** | **Ty3/Gypsy** | **Ty1/Copia** |  |
| Garlic | *Allium sativum* | 16.24 | 91 | 14.78 | 66 | 10.7 | 57.5 | 8.6 | (Sun et al., 2020) |
| Tausch's goatgrass | *Aegilops tauschii* | 4.54 | 85.9 | 3.7 | 58.24 | 2.51 | 38.3 | 16.5 | (Zhao et al., 2017) |
| Wheat | *Triticum aestivum* | 17 | 85 | 5.18 | 66.6 | 4.93 | 46 | 16.7 | (Wicker et al., 2018; Bariah et al., 2020) |
| Aloe vera | *Asphodelaceae* | 12.93 | 82.66 | 10.68 | 26.71 | 3.45 | 19.37 | 7.34 | (Jaiswal et al., 2021) |
| wheat | *Triticum turgidum* | 12 | 82.2 | 8.48 | 69.9 | 7.25 | 32.4 | 16.5 | (Avni et al., 2017) |
| Eelgrass | *Zostera marina* | 0.128 | 82.14 | 0.90 | 59.9 | 66.3 | 30.41 | 16.62 | (Zhang et al., 2020b) |
| Onion | *Allium cepa* | 14.9 | 81.86 | 12.3 | 72.24 | 10.2 | 6.33 | 2.98 | (Finkers et al., 2021) |
| Ginger | *Zingiber officinale* | 3.1 | 81.66 | 0.253 | 56.67 | 0.17 | 21.69 | 33.66 | (Cheng et al., 2021) |
| Wild wheat | *Triticum urartu* | 4.94 | 81.42 | 3.9 | 68.61 | 3.2 | 42.71 | 24.3 | (Ling et al., 2018) |
| Barley | *Hordeum vulgare* | 5.1 | 81.58 | 0.850 | 75.10 | 0.782 | 22.2 | 15.32 | (Langridge et al., 2012) |
| Maize | *Zea mays* | 2.16 | 79.44 | 0.177 | 75.08 | 0.170 | 37.73 | 21.75 | (Schnable et al., 2009) |
| Coconut palm | *Cocos nucifera* | 2.42 | 72.75 | 1.6 | 67.10 | 1.47 | 61 | 67 | (Xiao et al., 2017; Yang et al., 2021) |
| Lotus | *Nelumbo nucifera* | 0.929 | 72.34 | 0.296 | 44.75 | 0.183 | 17.61 | 15.74 | (Zhang et al., 2020b) |
| Rye | *Secale cereale* | 7.9 | 71.61 | 5.6 | 59.78 | 4.7 | 33.70 | 14.13 | (Bauer et al., 2017) |
| Jenkin's Rattan Palm | *Daemonorops jenkinsiana* | 1.61 | 70 | 1.12 | 61.09 | 0.982 | 41.05 | 24.04 | (Zhao et al., 2018) |
| Amborella | *Amborella trichopoda* | 0.660 | 70.11 | 0.287 | 45.74 | 0.187 | 16.29 | 21.07 | (Zhang et al., 2020b) |
| Giant Duckweed | *Spirodela polyrhiza* | 0.191 | 67.47 | 0.20 | 50.13 | 0.15 | 23.83 | 15.23 |  |
| Lily | *Nymphaea colorata* | 0.409 | 66.16 | 0.106 | 48.07 | 0.77 | 12.52 | 20.27 |  |
| Tianma | *Gastrodia elata* | 1.18 | 66.18 | 0.702 | 55.94 | 0.593 | 45.04 | 7.10 | (Yuan et al., 2018a) |
| Soybean | *Glycine max* | 1.11 | 61.47 | 0.587 | 42.24 | 0.403 | 29.52 | 12.47 | (Schmutz et al., 2010) |
| - | *Dendrobium catenatum* | 1.11 | 67.12 | 0.788 | 45.72 | 0.537 | 18.49 | 27.36 | (Zhang et al., 2016) |
| Aquatic model plant | *Lemna minor* | 0.481 | 61.46 | 0.299 | 31.20 | 0.152 | 10.59 | 18.79 | (Van Hoeck et al., 2015) |
| Red silk cotton tree | *Bombax ceiba* | 0.895 | 60.30 | 0.524 | 47.86 | 0.415 | 37.45 | 10.41 | (Gao et al., 2018) |
| Pearl millet | *Cenchrus americanus* | 1.58 | 60 | 1.22 | 50.1 | 1.0 | 28.8 | 22.2 | (Varshney et al., 2017) |
| Moth orchid | *Phalaenopsis aphrodite* | 1.02 | 60.3 | 0.763 | 59.15 | 0.625 | 49.9 | 10.6 | (Chao et al., 2018) |
| Agar wood | *Aquilaria agallocha* | 0.728 | 59.18 | 0.430 | 27.57 | 0.200 | 22.46 | 5.11 | (Chen et al., 2014) |
| Orchid | *Phalaenopsis equestris* | 1.16 | 59.6 | 0.964 | 46.4 | 0.504 | 39.66 | 6.95 | (Cai et al., 2015) |
| Moso bamboo | *Phyllostachys edulis* | 2.05 | 59 | 1.2 | 40.35 | 0.827 | 23.41 | 16.43 | (Zhou et al., 2017) |
| Moso bamboo | *Phyllostachys heterocycle* | 2.05 | 59 | 1.2 | 37.3 | 0.764 | 24.6 | 12.3 | (Peng et al., 2013) |
| Date palm | *Phoenix dactylifera* | 0.658 | 58 | 0.382 | 21.99 | 0.144 | 14.03 | 4.17 | (Faisal, 2019) |
| Seagrass Species | *Zostera muelleri* | 0.889 | 55.6 | 0.339 | 20.11 | 0.128 | 14.48 | 6.7 | (Lee et al., 2016) |
| Sorghum | *Sorghum bicolor* | 0.740 | 54.52 | 0.460 | 54.43 | 0.400 | 19.0 | 5.18 | (Paterson et al., 2009) |
| Durian | *Durio zibethinus* | 0.738 | 54.80 | 0.391 | 30 | 0.210 | 26.18 | 3.20 | (Teh et al., 2017) |
| Rattan | *Calamus simplicifolius* | 1.98 | 54.15 | 1.06 | 47.85 | 0.938 | 28.43 | 15.42 | (Zhao et al., 2018) |
| Garden asparagus | *Asparagus officinalis* | 17 | 53 | 9 | 28 | 4.76 | 24.4 | 4.1 | (Li et al., 2014) |
| Sugarcane | *Saccharum officinarum* | 0.800-0.900 | 53.6 | 0.428 | 40 | 320 | 24.8 | 15.25 | (Garsmeur et al., 2018) |
| Wild sugarcane | *S. spontaneum* | 0.800-0.900 | 46.5 | 372 | 34.6 | 0.276 | 22.34 | 12.09 |  |
| Sugarcane | *R570 cultivar* | 0.800-0.900 | 50.9 | 0.407 | 38.1 | 0.304 | 24.1 | 14 |  |
| Chickpea | *Cicer arietinum* | 0.738 | 49.41 | 0.258 | 45.64 | 0.238 | 19.75 | 18.45 | (Varshney et al., 2013) |
| Finger millet | *Eleusine coracana* | 1.19 | 49.92 | 0.728 | 33.2 | 0.325 | 25 | 8.02 | (Hittalmani et al., 2017) |
| Camphor | *Cinnamomum kanehirae* | 0.730 | 48.87 | 0.390 | 25.53 | 0.204 | 10.4 | 6.09 | (Chaw et al., 2019) |
| Saffron | *Crocus sativus* | 3.01 | 48.41 | 1.61 | 30.34 | 1 | 19.12 | 11.22 | (Ambardar et al., 2021) |
| Foxtail millet | *Setaria*  *Italica* | 0.423 | 46.4 | 0.126 | 31.6 | 0.85 | 22.1 | 7.2 | (Oliver et al., 2013) |
| Guinea yam | *Dioscorea rotundata* | 0.374 | 46.07 | 0.274 | 22.82 | 0.135 | 20.22 | 2.31 | (Tamiru et al., 2017) |
| Wild rice | *O. rufipogon* | 0.387.78 | 45.18 | 0.167 | 17.46 | 0.64 | 10.31 | 3.71 | (Li et al., 2020) |
| Wild rice | *O. longistaminata* | 0.392 | 36.69 | 0.109 | 12.96 | 0.38 | 7.48 | 2.09 |  |
| Wild rice | *Nipponbare*  *(MSU v7.0)* | 0.389 | 50.97 | 0.194 | 25.17 | 0.96 | 15.8 | 5.18 |  |
| White poplar | *Populus alba* | 0.467 | 44.61 | 0.200 | 20.45 | 0.92 | 10.45 | 10 | (Ma et al., 2019) |
| Euphrates poplar | *P. euphratica* |  | 42.78 | 0.212 | 36.69 | 0.182 | 4.68 | 12.01 |  |
| Black cottonwood | *P. trichocarpa* |  | 46.33 | 0.201 | 33.05 | 0.143 | 15.04 | 18.01 |  |
| Rice | *Oryza sativa vg. japonica* | 0.389 | 49 | 0.196 | 23.52 | 0.94 | 19.44 | 4.08 | (Stein et al., 2018) |
| Rice | *O. sativa vg. indica [93-11]* | 0.466 | 43.06 | 0.207 | 19.43 | 0.93 | 15.87 | 3.56 |  |
| Rice | *O. sativa vg. indica [IR 8]* | 0.389 | 36.99 | 0.148 | 25.02 | 0.100 | 21.89 | 3.13 |  |
| Rice | *O. sativa vg. aus [N 22]* | 0.362 | 46.32 | 0.174 | 22.43 | 0.84 | 18.77 | 3.66 |  |
| Rice | *O. nivara* | 0.338 | 36.73 | 0.157 | 13.64 | 0.58 | 10.56 | 3.07 |  |
| Rice | *O. barthii* | 0.308 | 38.48 | 0.137 | 14.68 | 0.52 | 11.36 | 3.32 |  |
| Rice | *O. glumaepatula* | 0.373 | 31.51 | 0.143 | 12.44 | 0.56 | 9.12 | 2.57 |  |
| Rice | *O. meridionalis* | 0.336 | 27.49 | 0.119 | 7.5 | 0.32 | 7.5 | 0.01 |  |
| Rice | *O. punctata* | 0.394 | 49.94 | 0.204 | 29.3 | 0.120 | 24.13 | 5.17 |  |
| Rice | *O. brachyanth* | 0.362 | 28.76 | 0.110 | 9.12 | 0.35 | 5.98 | 3.14 |  |
| Rice | *Leersia perrieri*  *Outgroup* | 0.267 | 26.83 | 0.88 | 12.23 | 0.40 | 8.32 | 3.91 |  |
| Rice | *O. brachyantha* | 0.261 | 29.17 | 0.70 | 10.47 | 0.25 | 3.09 | 8.74 | (Chen et al., 2013) |
| Rice | *Oryza sativa ssp. indica* | 0.466 | 40.41 | 0.194 | 33.11 | 0.154 | 23.28 | 10.72 | (Yu et al., 2002) |
| Japanese zoysiagrass | *Zoysia japonica 'Nagirizaki'* | 0.235 | 40.9 | 0.136 | 9.5 | 0.131 | 6.0 | 3.5 | (Tanaka et al., 2016) |
| Manila grass | *Z. matrella 'Wakaba'* | 0.227 | 45.9 | 0.258 | 10.1 | 0.57 | 6.5 | 3.6 |  |
| Mascarenegrass | *Z. pacifica 'Zanpa'* | 0.234 | 44.7 | 7.3 | 10.6 | 0.41 | 6.7 | 3.8 |  |
| Banana | *Musa acuminata* | 0.462 | 38.95 | 0.179 | 34.07 | 0.157 | 16.2 | 16.71 | (Wu et al., 2016) |
| Banana | *Musa balbisiana* | 0.402 | 28.85 | 0.108 | 24.11 | 0.94 | 18.8 | 6.3 | (Davey et al., 2013) |
| Banana | *Musa schizocarpa* | 0.587 | 59.95 | 0.357 | 27.96 | 0.166 | 17.06 | 10.9 | (Belser et al., 2018) |
| Desiccation tolerant grass | *Oropetium thomaeum* | 0.245 | 43.80 | 0.108 | 25.50 | 0.107 | 21.8 | 36.9 | (Hittalmani et al., 2017) |
| Barnyardgrass | *Echinochloa crus-galli* | 1.27 | 40.68 | 0.567 | 21.91 | 0.30 | 18.81 | 3.1 | (Guo et al., 2017) |
| *-* | *Apostasia shenzhenica* | 0.471 | 40.05 | 0.146 | 22.06 | 0.76 | 11.84 | 4.97 | (Zhang et al., 2017a) |
| Chinese wild rice | *Zizania latifolia* | 0.604 | 37.65 | 0.227 | 29.80 | 0.180 | 13.20 | 15.97 | (Guo et al., 2015) |
| Ryegrass | *Lolium perenne* | 2.06 | 28.82 | 1.15 | 27.43 | 1.09 | 20.33 | 7.05 | (Byrne et al., 2015) |
| Tef | *Eragrostis tef* | 0.730 | 27.46 | 0.200 | 14.96 | 0.109 | 1.40 | 2.67 | (Gebre et al., 2016) |
| Rice | *Oryza sativa* | 0.420 | 25.78 | 0.168 | 23.47 | 0.109 | 12.03 | 2.47 | (Zhang and Gao, 2017) |
| Wild grass | *Brachypodium distachyon* | 0.270 | 23.33 | 0.125 | 21.29 | 0.122 | 16.05 | 4.86 | (Initiative, 2010) |
| African oil palm | *Elaeis guineensis* | 1.53 | 18 | 0.282 | 6 | 0.94 | 0.12 | 5 | (Singh et al., 2013) |

| **Dicots** | | | | | | | | | |
| --- | --- | --- | --- | --- | --- | --- | --- | --- | --- |
| **Common name** | **Scientific name** | **Total**  **genome**  **Size**  **(Gbp)** | **Total TE % in genome** | | **Total % of LTR retrotransposons in genome** | | **Total % of super families in LTR-retrotransposons** | | **Reference** |
|  |  |  | **%** | **Gbp** | **%** | **Gbp** | **Ty3/**  **Gypsy** | **Ty1/**  **Copia** |  |
| Pepper | *Capsicum annuum* | 3.5 | 91.54 | 0.772 | 89.32 | 0.753 | 87.77 | 3.54 | (de Assis et al., 2020) |
| Pepper | *C. chinense* |  | 98.83 | 0.509 | 98.78 | 0.509 | 98.43 | 0.33 |  |
| Pepper | *C. baccatum* |  | 72.06 | 0.573 | 70.55 | 0.561 | 63.27 | 6.7 |  |
| Tobacco | *Nicotiana attenuata* | 2.5 | 81.0 | 1.69 | 66.68 | 1.33 | 57.42 | 6.26 | (Xu et al., 2017b) |
| Tobacco | *N. obtusifolia* | 1.5 | 64.8 | 0.792 | 45.98 | 0.562 | 42.5 | 3.48 |  |
| Cotton | *Gossypium arboreum* | 2.22 | 82.03 | 1.40 | 79.96 | 1.37 | 64.13 | 7.21 | (Cheng et al., 2019) |
| Cotton | *G. raimondii* |  | 62.44 | 0.475 | 58.70 | 0.446 | 38.12 | 3.56 |  |
| Cotton | *G. hirsutum (A-genome)* |  | 58.95 | 0.876 | 57.07 | 0.848 | 44.8 | 6.67 |  |
| Cotton | *G. hirsutum (D-genome)* |  | 53.91 | 0.450 | 50.3 | 0.420 | 32.52 | 11.14 |  |
| Cotton | *G.barbadense* | 2.47 | 34.85 | 0.485 | 26.6 | 0.384 | 21.5 | 5.1 | (Liu et al., 2015) |
| Papaya | *Carica papaya* | 0.372 | 83.6 | 0.180 | 68.8 | 0.148 | 52.8 | 10.4 | (Wu et al., 2013) |
| Sunflower | *Helianthus annuus* | 3.5 | 81 | 1.98 | 77.75 | 1.9 | 59.93 | 19.83 | (Staton et al., 2012) |
| Miers | *Jaltomata sinuosa* | 1.45 | 80.29 | 1.15 | 50.1 | 1.04 | 48.72 | 2.34 | (Wu et al., 2019b) |
| Tree peony | *Paeonia suffruticosa* | 13.79 | 80.24 | 11 | 49.29 | 6.87 | 38.91 | 5.12 | (Lv et al., 2020) |
| Common tobacco | *Nicotiana tabacum* | 4.57-4.6 | 72.7-78.9 | 2.58-2.85 | 33-35.7 | 1.19-1.28 | 20-20.1 | 15.6 | (Sierro et al., 2014) |
| Common tobacco | *N. tomentosiformis* | 2.36 | 74.845 | 1.26 | 33.75 | 0.570 | 20-20.32 | 13.43 | (Sierro et al., 2013) |
| Pea | *Pisum sativum* | 4.45 | 77.78 | 2.45 | 72.72 | 2.29 | 38.88 | 12.0 | (Kreplak et al., 2019) |
| Pitcher plant | *Cephalotus follicularis* | 1.61 | 77.42 | 1.24 | 75.57 | 1.22 | 64.46 | 11.11 | (Fukushima et al., 2017) |
| Resurrection plant | *Boea hygrometrica* | 1.69 | 75.75 | 1.17 | 18.44 | 0.285 | 9.58 | 8.68 | (Xiao et al., 2015) |
| Marijuana | *Cannabis sativa* | 0.808 | 74.75 | 0.607 | 50 | 0.406 | 34.88 | 15.12 | (Gao et al., 2020) |
| Spinach | *Spinacia oleracea* | 0.989 | 74.43 | 0.618 | 52.1 | 0.439 | 27.55 | 25.47 | (Xu et al., 2017a) |
| Lettuce | *Lactuca sativa* | 2.38 | 74.2 | 1.8 | 61.5 | 1.5 | 33.9 | 24.9 | (Reyes-Chin-Wo et al., 2017) |
| Tea tree | *Camellia sinensis CSS-BY* | 3.25 | 74.13 | 0.216 | 38.91 | 0.113 | 34.11 | 4.8 | (Zhang et al., 2020c) |
| Tea tree | *Camellia sinensis CSA-YK10* | 3.02 | 69.35 | 0.208 | 46.59 | 0.139 | 40.39 | 6.20 |  |
| Tea tree | *Camellia sinensis CSS-SCZ* | 2.98 | 64.77 | 0.200 | 54.09 | 0.167 | 45.85 | 8.24 |  |
| Tobacco | *Nicotiana sylvestris* | 2.68 | 71.95 | 1.0 | 29.88 | 0.666 | 20-20.32 | 13-13.43 | (Sierro et al., 2013) |
| Buckwheat | *Fagopyrum esculentum* | 1.34 | 71.43 | 0.608 | 14.22 | 0.103.5 | 12.15 | 2.07 | (Yasui et al., 2016) |
| Rubber tree | *Hevea brasiliensis* | 1.47 | 70.82 | 1.42 | 65.88 | 0.969 | 39.79 | 12.73 | (Rahman et al., 2013; Liu et al., 2020b) |
| Peanut | *Arachis hypogaea* | 2.7 | 74.03 | 1.99 | 61.83 | 1.66 | 39.93 | 4.72 | (Bertioli et al., 2019) |
| Peanut | *Arachis ipaensis* |  | 68.5 | 0.861 | 45.72 | 0.574 | 18.6 | 2.98 | (Bertioli et al., 2016) |
| Peanut | *Arachis duranensis* |  | 61.73 | 0.580.2 | 44.74 | 0. 420 | 18.14 | 3.17 |  |
| Eutrema | *E. yunnanense* | 0.442 | 70 | 0.311.4 | 31.3 | 0.139 | 22.2 | 9.1 | (Guo et al., 2018) |
| Eutrema | *E. heterophyllum* | 0.360 | 67 | 0.243 | 28.15 | 0.102 | 18.5 | 9.51 |  |
| Sugar beets | *Beta vulgaris* | 0.758 | 70.91 | 0.196 | 43.64 | 0.120 | 16.21 | 12.05 | (Wu et al., 2013) |
| Chrysanthemum | *Chrysanthemum nankingense* | 2.53 | 69.58 | 1.75 | 47.10 | 1.19 | 21.54 | 25.40 | (Song et al., 2018) |
| Wheel tree | *Trochodendron aralioides* | 1.07 | 68.91 | 0.764 | 60.6 | 0.672 | 33.72 | 20.76 | (Li et al., 2021a) |
| Wheel tree | *Tetracentron sinense* |  | 51.09 | 0.503 | 41 | 0.404 | 22 | 13.74 |  |
| Eggplant | *Solanum melongena* | 1.155 | 68.9 | 0.785.4 | 63.9 | 0.738 | 52 | 7.9 | (Hirakawa et al., 2014; Li et al., 2019a) |
| Hazelnut | *Corylus mandshurica* | 0.367 | 68.74 | 0.252 | 57.92 | 0.212 | 17.46 | 16.98 | (Li et al., 2021b) |
| Russian dandelion | *Taraxacum kok-saghyz* | 1.29 | 68.56 | 0.875 | 40.73 | 0.520 | 19.79 | 20.39 | (Lin et al., 2018) |
| Olive | *Olea europaea* | 1.3 | 67.37 | 0.743 | 56.88 | 0.627 | 20.54 | 20.2 | (Rao et al., 2021) |
| Pink Barren Strawberry | *Potentilla micrantha* | 0.327 | 67.5 | 0.275 | 24.1 | 0.98.5 | 16.5 | 7.4 | (Buti et al., 2018) |
| Grape | *Vitis vinifera* | 0.470 | 66.64 | 172 | 52.21 | 134 | 27.41 | 16.02 | (Wu et al., 2013) |
| *-* | *Malania oleifera* | 1.51 | 65.45 | 0.427 | 58.23 | 0.385 | 28.15 | 29.51 | (Xu et al., 2019) |
| Pitaya | *Hylocereus undatus* | 1.41 | 64.8 | 0.881 | 48.83 | 664.19 | 60.83 | 37.89 | (Jian-ye et al., 2021) |
| Opium poppy | *Papaver somniferum* | 2.72 | 64.53 | 1.69 | 54.40 | 1.42 | 30.1 | 24.1 | (Pei et al., 2021) |
| Morning Glory | *Ipomoea nil* | 0.734 | 64.5 | 0.474 | 23.5 | 0.172 | 15.3 | 8.2 | (Sun et al., 2018) |
| Rose | *Rosa chinensis* | 0.518 | 63.21 | 0.327 | 28.33 | 0.146 | 15.70 | 12.63 | (Saint-Oyant et al., 2018) |
| Tomato | *Solanum lycopersicum* | 0.900 | 63.2 | 0.466 | 62.3 | 0.459 | 19.7 | 6.3 | (Tomato Genome Consortium, 2012; Domínguez et al., 2020) |
| Petunia | *Petunia axillaris* | 1.26 | 63.08 | 0.655 | 40.41 | 0.292 | 27.39 | 13.02 | (Bombarely et al., 2016) |
| Castor bean | *Ricinus communis* | 0.320 | 61.4 | 0.230 | 36.07 | 0.61 | 22.75 | 9.48 | (Chan et al., 2010) |
| Tuliptree | *Liriodendron tulipifera* | 1.75 | 61.64 | 1.07 | 53.53 | 0.931 | 40.45 | 13.08 | (Chen et al., 2019) |
| Apple  HFTH1 | *Malus domestica Borkh* | 0.660 | 63.17 | 0.416 | 46.15 | 0.304 | 25.41 | 15.86 | (Zhang et al., 2019) |
| Apple  GDDH13 | *Malus domestica Borkh* | 0.643 | 61.55 | 0.384 | 43.97 | 0.274 | 24.57 | 14.42 |  |
| Norway spruce | *Picea abies* | 20 | 69.53 | 13.9 | 58.33 | 56.3 | 35.38 | 16.13 | (Nystedt et al., 2013) |
| Scots pine | *Pinus sylvestris* | 22.4 | 52.26 | 11.7 | 23.39 | 5.23 | 15.80 | 7.38 |  |
| white spruce | *Picea glauca* | 19.7 | 60.7 | 11.9 | 46.49 | 9.1 | 31.38 | 9.72 |  |
| Siberian fir | *Abies sibirica* | 15.5 | 53.9 | 8.3 | 22.06 | 3.39 | 3.56 | 8.01 |  |
| Juniper | *Juniper communis* | 11.7 | 46.25 | 5.41 | 17.92 | 2.09 | 6.83 | 10.90 |  |
| European Yew | *Taxus baccata* | 11.2 | 50.46 | 5.6 | 22.70 | 2.51 | 19.81 | 2.80 |  |
| Melinjo | *Gnetum gnemon* | 3.7 | 62.84 | 2.32 | 24.98 | 0.922 | 22.41 | 2.09 |  |
| Sweet sagewort | *Artemisia annua* | 1.74 | 61.57 | 1.01 | 22.69 | 0.373 | 19.35 | 3.34 | (Shen et al., 2018) |
| Hardy rubber tree | *Eucommia ulmoides* | 1.2 | 61.24 | 0.723 | 30.63 | 0.361 | 16.7 | 13.22 | (Wuyun et al., 2018) |
| White lupin | *Lupinus albus* | 0.451 | 60.52 | 0.272 | 34 | 0.153 | 21.56 | 9.24 | (Hufnagel et al., 2020) |
| Wild tomato | *Solanum pennellii* | 0.942 | 60.6 | 0.644 | 45 | 0.428 | 37 | 8.3 | (Bolger et al., 2014) |
| Cranberry | *Vaccinium macrocarpon* | 0.492 | 50.49 | 0.255 | 14.01 | 0.70 | 8.32 | 4.99 | (Diaz-Garcia et al., 2021) |
| Cranberry | *V. microcarpum* |  | 50.31 | 0.313 | 14.86 | 92.5 | 8.68 | 4.98 |  |
| Globe artichoke | *Cynara cardunculus* | 1.07 | 58.4 | 0.424 | 41.73 | 0.314 | 16.48 | 27.84 | (Scaglione et al., 2016) |
| Dodders | *Cuscuta australis* | 0.272 | 58 | 0.155 | 23.9 | 0.63 | 10.4 | 15.5 | (Sun et al., 2018) |
| *-* | *Petunia inflata* | 1.29 | 59.22 | 0.597 | 36.98 | 0.382 | 25.67 | 11.32 | (Bombarely et al., 2016) |
| Wild Tomato | *Solanum pimpinellifolium* | 0.739 | 59.5 | 0.439 | 42.15 | 0.325 | 37.7 | 5.8 | (Razali et al., 2018) |
| Saguaro cactus | *Carnegiea gigantea* | 0.980 | 57.67 | 0.554 | 26.12 | 0.201 | 22.52 | 4.72 | (Copetti et al., 2017) |
| Rose | *Hibiscus syriacus* | 1.75 | 57.61 | 1.0 | 27.8 | 0.527 | 20.2 | 7.6 | (Kim et al., 2017) |
| Scarlet sage | *Salvia splendens* | 0.711 | 57.52 | 0.465 | 26.49 | 0.214 | 18.15 | 7.92 | (Dong et al., 2018) |
| Canola | *Brassica napus* species | 1.2 -1.27 | 56.85-58.2 | 0.55.6-0.56.6 | 31.3-32.9 | 0.30-0.32 | 15.8-16.7 | 12.7-13.6 | (Song et al., 2020) |
| Cultivated Potato | *Solanum chacoense* | 0.882 | 56.85 | 0.501 | 40.03 | 0.356 | 27.12 | 13.17 | (Leisner et al., 2018) |
| Cauliflower | *Brassica oleracea* | 0.584 | 56.65 | 0.331 | 32.71 | 0.191 | 21.07 | 3.9 | (Sun et al., 2019a) |
| Asiatic pennywort | *Centella asiatica* | 0.430 | 56.38 | 0.242 | 35.75 | 0.149 | 12.22 | 22.66 | (Pootakham et al., 2021) |
| Black raspberry | *Rubus occidentalis* | 0.243 | 56.6 | 0.136 | 22.2 | 0.53 | 11.6 | 10.6 | (VanBuren et al., 2016) |
| Wild rose | *Rosa multiflora* | 0.711 | 56.4 | 0.417 | 17.2 | 0.126 | 3.7 | 6.6 | (Nakamura et al., 2018) |
| Meyer | *Panax ginseng* | 3.43 | 56.11 | 1.92 | 51.91 | 1.78 | 42.82 | 8.28 | (Scaglione et al., 2016) |
| Jute | *Corchorus olitorius* | 0.447 | 53.72 | 0.110 | 49.73 | 0.87 | 19.13 | 30.6 | (Islam et al., 2017) |
| Jute | C. capsularis | 0.404 | 56.17 | 0.115 | 51.94 | 0.106 | 20 | 31.9 |  |
| Monkeyflower | *Mimulus guttatus* | 0.289 | 55.77 | 0.161 | 28.13 | 0.81 | 22.08 | 6.05 | (He et al., 2018) |
| Watermelon | *Citrullus lanatus* | 0.362.7 | 55.55 | 0.202 | 28.25 | 0.103 | 19.86 | 8.39 | (Guo et al., 2019) |
| Apple-ring acacia | *Faidherbia albida* | 0.653 | 54.8 | 0.358 | 44.6 | 0.291 | 23.05 | 21.0 | (Chang et al., 2019) |
| Durango root | *Datisca glomerata* | 0.827 | 54.68 | 0.117 | 38.09 | 0.81 | 4.44 | 12.55 | (Griesmann et al., 2018) |
| Lupin | *Lupinus angustifolius* | 0.609 | 54.37 | 0.331 | 28.23 | 0.172 | 20.11 | 8.12 | (Hane et al., 2017) |
| Potato | *Solanum tuberosum* | 0.840 | 54.4 | 0.318 | 53.2 | 0.311 | 15.2 | 3.8 | (Tomato Genome Consortium, 2012) |
| Wild radish genomes | *Raphanus raphanistrum* | 0.418-0.514 | 46.51-53.94 | 0.194-0.268 | 24.51-34.80 | 0.102-169 | 9.67-13.02 | 13.57-23.51 | (Zhang et al., 2021) |
| Bitterweed | *Andrographis paniculata* | 0.280 | 53.26 | 0.143 | 19.58 | 0.52 | 10.54 | 8.42 | (Sun et al., 2019b) |
| Oak | *Quercus robur* | 0.716.6 | 53.3 | 0.420 | 24.65 | 0.194 | 13.61 | 11.04 | (Plomion et al., 2018) |
| Pear | *Pyrus bretschneideri* | 0.512 | 53.1 | 0.271 | 42.98 | 0.220 | 25.48 | 16.88 | (Wu et al., 2013) |
| Longan | *Dimocarpus longan* | 0.445 | 52.87 | 0.261 | 36.54 | 0.180 | 25.32 | 11.22 | (Lin et al., 2017) |
| Fleabane | *Erigeron breviscapus* | 1.2 | 52.65 | 0.640 | 37.82 | 0.460 | 17.51 | 19.31 | (Yang et al., 2017b) |
| *-* | *Rhododendron delavayi* | 0.695 | 51.77 | 0.359 | 37.48 | 0.260 | 25.49 | 6.84 | (Zhang et al., 2017c) |
| Pomegranate | *Punica granatum* | 0.336 | 51.6 | 0.140 | 17.42 | 0.47 | 11.55 | 5.87 | (Yuan et al., 2018b) |
| Pigeonpea | *Cajanus cajan* | 0.833 | 51.67 | 0.313 | 19.18 | 0.116 | 11.79 | 6.22 | (Varshney et al., 2012) |
| Pineapple | *Ananas comosus* | 0.526 | 51.48 | 0.198 | 31.68 | 0.121 | 24.38 | 7.30 | (Ming et al., 2015) |
| Cucurbit | *Siraitia grosvenorii* | 0.469.5 | 51.14 | 0.240 | 14.38 | 0.67 | 11.79 | 6.22 | (Xia et al., 2018) |
| Tartary buckwheat | *Fagopyrum tataricum* | 0.489 | 50.96 | 0.249 | 38.69 | 0.189 | 30.52 | 5.58 | (Zhang et al., 2017b) |
| Medicinal herb | *Rhodiola crenulate* | 0.344 | 50.81 | 0.175 | 38.78 | 0.133 | 31.64 | 7.14 | (Fu et al., 2017) |
| Persian walnut | *Juglans regia* | 0.606 | 50.35 | 0.305 | 35.38 | 0.214 | 8.40 | 6.57 | (Martínez‐García et al., 2016) |
| Eucalyptus | *Eucalyptus grandis* | 0.640 | 50 | 0.320 | 44.5 | 0.284 | 26.3 | 18.2 | (Myburg et al., 2014) |
| Cassava | *Manihot esculenta* | 0.751 | 50.34 | 0.293 | 31.97 | 0.186 | 27.86 | 4.17 | (Bredeson et al., 2016) |
| Morning glory | *Ipomoea triloba* | 0.495.9 | 50.03 | 0.231 | 0.42 | 0.9 | 0.25 | 0.17 | (Wu et al., 2018) |
| Arabidopsis | *Arabidopsis thaliana* (TAIR10) | 0.135 | 49.33 | 0.12 | 34.66 | 0.8 | 24.2 | 5.08 | (Pereira, 2004; Zhang et al., 2020b) |
| Coffee | *Coffea canephora* | 0.710 | 49.2 | 0.371 | 42 | 0.317 | 24.1 | 6.84 | (Denoeud et al., 2014) |
| Plum | *Prunus salicina* | 0.311.8 | 48.28 | 0.137 | 42.10 | 0.119 | 26.1 | 16.0 | (Liu et al., 2020a) |
| Amaranth | *Amaranthus hypochondriacus* | 0.377 | 47.7 | 0.179 | 10.94 | 0.41 | 3.85 | 6.80 | (Clouse et al., 2016) |
| Wild flowering cherry | *Prunus yedoensis* | 0.323 | 47.23 | 0.150 | 22.75 | 72.6 | 13.12 | 8.25 | (Baek et al., 2018) |
| Bottle gourd | *Lagenaria siceraria* | 0.313.4 | 46.93 | 0.139 | 17.18 | 32.4 | 6.29 | 10.89 | (Wu et al., 2017) |
| Mungbean | *Vigna radiata* | 0.579 | 46.86 | 0.176 | 42.1 | 158.2 | 21.30 | 11.32 | (Kang et al., 2014) |
| Guava | *Psidium guajava* | 0.443.8 | 46.32 | 0.205 | 38.23 | 169 | 22.11 | 16.2 | (Feng et al., 2021) |
| Dodder | *Cuscuta campestris* | 0.477 | 46.2 | 0.220 | 44.5 | 212 | 16.9 | 34.1 | (Vogel et al., 2018) |
| Carrot | *Daucus carota* | 0.473 | 46 | 0.193 | 27.4 | 115.6 | 21.5 | 37.7 | (Iorizzo et al., 2016) |
| Sweet potato | *Ipomoea batatas* | 4.4 | 45.6 | 0.382 | 10.98 | 0.92 | 7.32 | 3.67 | (Yang et al., 2017a) |
| Jasmine | *Jasminum sambac* | 0.550 | 45.56 | 0.259 | 33.97 | 0.193 | 15.0 | 16.2 | (Chen et al., 2020) |
| Common bean | *Phaseolus vulgaris* | 0.473 | 45.42 | 0.214 | 36.66 | 0.173 | 25.12 | 9.37 | (Schmutz et al., 2014) |
| White poplar | *Populus alba* | 0.464 | 44.61 | 0.200 | 20.45 | 0.92 | 10.45 | 10 | (Ma et al., 2019) |
| Euphrates poplar | *P. euphratica* |  | 42.78 | 0.211 | 36.69 | 0.182 | 14.68 | 12.01 |  |
| Euphrates Poplar | *P. trichocarpa* |  | 46.33 | 0.201 | 33.05 | 0.143 | 15.04 | 18.01 |  |
| Clementine | *Citrus clementina* | 0.301.4 | 44.67 | 0.134 | 20 | 0.60 | 12.01 | 7.88 | (Wu et al., 2014) |
| Sesame | *Sesamum indicum* | 0.270 | 44.59 | 0.120 | 18.20 | 0.49 | 15.18 | 3.02 | (He et al., 2018) |
| Subterranean clover | *Trifolium subterraneum* | 0.471 | 44.4 | 0.216 | 9.0 | 0.43 | 3.5 | 5.4 | (Hirakawa et al., 2016) |
| Mei | *Prunus mume* | 0.280 | 44.1 | 0.104 | 18.6 | 0.44 | 8.6 | 10 | (Zhang et al., 2012) |
| Marula | *Sclerocarya birrea* | 0.330 | 45.18 | 0.149 | 38.78 | 0.128 | 28.2 | 10.58 | (Chang et al., 2019) |
| Wild sweet Poatato | *Ipomoea trifida* | 0.526 | 44.15 | 0.217 | 10.9 | 0.52 | 5.42 | 5.48 | (Wu et al., 2018) |
| Kiwifruit | *Actinidia chinensis* | 0.653 | 43.42 | 0.270 | 23.38 | 0.152 | 18.35 | 5.03 | (Wu et al., 2019a) |
| Box orange | *Atalantia buxifolia* | 0.328 | 43.55 | 0.137 | 24.15 | 0.126 | 12.71 | 12.79 | (Wang et al., 2017) |
| Pomelo | *Citrus grandis* | 0.380 | 45.83 | 0.158 | 26.66 | 0.93 | 15.62 | 11.4 |  |
| Papeda | *C. ichangensis* | 0.391 | 39.31 | 0.132 | 15.15 | 0.51 | 7.61 | 8.89 |  |
| Citron | *C. medica* | 0.406 | 43.80 | 0.164 | 25.16 | 0.99 | 13.74 | 12.92 |  |
| Mandarin | *Citrus reticulata* | 0.370 | 50.5 | 0.334 | 47.7 | 0.316 | 25.25 | 22.5 | (Wang et al., 2018) |
| Medicinal plant | *Pogostemon cablin* | 1.76 | 43.68 | 0.770 | 27.76 | 0.489 | 24.23 | 3.43 | (He et al., 2018) |
| Adzuki bean | *Vigna angularis* | 0.538 | 43.09 | 0.172 | 28.94 | 0.115 | 18.98 | 9.96 | (Kang et al., 2015) |
| Ramie | *Boehmeria nivea* | 0.341 | 43.7 | 0.150 | 33.8 | 0.116 | 26.1 | 7.7 | (Luan et al., 2018) |
| Sweet cherry | *Prunus avium* | 0.273 | 43.8 | 0.119 | 6.0 | 0.15 | 2.9 | 3.1 | (Shirasawa et al., 2017) |
| Bitter gourd | *Momordica charantia* | 0.285.5 | 42.75 | 0.122 | 11.9 | 0.34 | 4.8 | 6.8 | (Xia et al., 2018) |
| Red clover | *Trifolium pratense* | 0.309 | 41.82 | 0.129 | 20.57 | 0.63 | 2.45 | 7.89 | (De Vega et al., 2015) |
| Medicinal plant | *Macleaya cordata* | 0.378 | 41.73 | 0.157 | 27.74 | 0.104 | 13.57 | 9.87 | (Liu et al., 2017) |
| Schott's yellowhood | *Nissolia schottii* | 0.471 | 41.68 | 0.896 | 18.74 | 0.45 | 4.03 | 1.85 | (Griesmann et al., 2018) |
| Cacao tree | *Theobroma cacao* | 0.346 | 41.53 | 0.137 | 31.98 | 0.105 | 27.14 | 4.86 | (Motamayor et al., 2013) |
| Mulberry | *Morus notabilis* | 0.330 | 41.3 | 0.127 | 13.44 | 0.43 | 27.14 | 4.86 | (He et al., 2013) |
| Horse radish | *Moringa oleifera* | 0.217 | 40.57 | 0.87 | 22.69 | 0.49 | 12.6 | 10.09 | (Chang et al., 2019) |
| Mango | *Mangiferaindica* | 0.392.9 | 40.54 | 0.159 | 16.17 | 0.63 | 4.74 | 9.85 | (Wang et al., 2020) |
| Field mustard | *Brassica rapa* | 0.485 | 39.51 | 0.191 | 27.14 | 0.131 | 3.11 | 2.82 | (Wang et al., 2011) |
| Perennial model plant | *Arabis alpina* | 0.375 | 39.46 | 0.148 | 13.52.70 | 0.50 | 9.64 | 3.88 | (Willing et al., 2015) |
| Almond | *Prunus dulcis* | 0.238 | 39.2 | 0.228 | 21.28 | 0.123 | 9.63 | 10.65 | (Alioto et al., 2020) |
| Jujube | *Ziziphus jujuba* | 0.351 | 39.34 | 0.138 | 38.81 | 0.136 | 14.33 | 11.3 | (Huang et al., 2016) |
| Fuchsia Begonia | *Begonia fuchsioides* | 0.935 | 38.92 | 0.60 | 22.21 | 0.34 | 5.09 | 2.16 | (Griesmann et al., 2018) |
| London rocket | *Sisymbrium irio* | 0.259 | 38.4 | 0.295 | 10.8 | 0.61 | 6.4 | 4.4 | (Haudry et al., 2013) |
| **-** | *Aethionema arabicum* | 0.203 | 37.1 | 0.242 | 10.4 | 0.46 | 5.3 | 5.2 |  |
| Saltwater cress | *Eutrema salsugineum* | 0.243 | 49.7 | 0.152 | 27 | 0.46 | 17.2 | 9.9 |  |
| Alabama gladecress | *Leavenworthia alabamica* | 0.174 | 26.5 | 0.129 | 7.3 | 0.20 | 3.3 | 4.0 |  |
| Lyrate Rockcress | *A. lyrata* | 0.207 | 31.6 | 0.120 | 13.1 | 0.19 | 9.8 | 3.2 |  |
| Capsella | *Capsella rubella* | 0.135 | 18.3 | 0.65 | 4.7 | 0.9 | 2.7 | 2.0 |  |
| Saltwater cress | *Schrenkiella parvula* | 0.114 | 13.3 | 0.43.4 | 4.1 | 0.7 | 2.0 | 2.1 |  |
| Spider flower | *Tarenaya hassleriana* | 0.290 | 43.35 | 0.110 | 38.19 | 0.97 | 29.11 | 9.08 | (Cheng et al., 2013) |
| Bambara groundnut | *Vigna subterranea* | 0.535 | 38.35 | 0.205 | 19.77 | 0.105 | 10.67 | (9.1 | (Chang et al., 2019) |
| Actinorhizal plant | Discaria *trinervis* | 0.649 | 38.17 | 0.53 | 16.31 | 0.22 | 6.61 | 2.16 | (Griesmann et al., 2018) |
| Hyacinth bean | *Lablab purpureus* | 0.395 | 37.18 | 0.147 | 23.78 | 0.94 | 13.6 | 10.18 | (Chang et al., 2019) |
| Cucumber | *Cucumis sativus* | 0.350 | 37.7 | 0. 85 | 12.16 | 0.27 | 5.29 | 6.87 | (Li et al., 2019b) |
| Pink Ipê | *Handroanthus impetiginosus* | 0.503 | 37.5 | 0.209 | 12.8 | 0.71 | 4.9 | 3.22 | (Silva-Junior et al., 2018) |
| primrose | *Primula vulgaris* | 0.411 | 37.03 | 0.152 | 13.21 | 0.16 | 3.05 | 9.70 | (Cocker et al., 2018) |
| Peach | *Prunus persica* | 0.265 | 37.14 | 0.84 | 19.56 | 0.44 | 9.97 | 8.6 | (Verde et al., 2013) |
| Red bayberry | *Morella rubra* | 0.313 | 36.4 | 0.114 | 21.4 | 0.67 | 9.9 | 5.1 | (Jia et al., 2019) |
| Strawberry | *Fragaria ananassa* | 0.813.4 | 36.08 | 0.270 | 28.87 | 0.232 | 11.94 | 2.69 | (Edger et al., 2019) |
| Medicinal legume | *Glycyrrhiza uralensis* | 0.379 | 35.92 | 0.135 | 9.8 | 0.35 | 4.48 | 4.84 | (Mochida et al., 2017) |
| European ash | *Fraxinu*s *excelsior* | 0.877 | 35.95 | 0.311 | 24.8 | 0.214 | 11.57 | 12.6 | (Sollars et al., 2017) |
| Partridge pea | *Chamaecrista fasciculata* | 0.550 | 34.76 | 0.34 | 18.87 | 0.17 | 1.59 | 8.19 | (Griesmann et al., 2018) |
| Alder | *Alnus glutinosa* | 0.461 | 34.33 | 0.120 | 13.08 | 0. 84 | 2.16 | 1.68 | (Griesmann et al., 2018) |
| Hop | *Humulus lupulus* | 2.57 | 34.67 | 0.710 | 32.2 | 0.66 | 12.7 | 4.2 | (Natsume et al., 2015) |
| Pyrethrum | *Tanacetum cinerariifolium* | 7.1 | 33.84 | 0.2 | 23.17 | 0.1 | 9.81 | 13.30 | (Yamashiro et al., 2019) |
| Eastern redbud | *Cercis canadensis* | 0.300 | 33.84 | 0.38 | 19.82 | 0.22 | 5.08 | 2.00 | (Griesmann et al., 2018) |
| Carnation | *Dianthus caryophyllus* | 0.622 | 33 | 0.165 | 7.3 | 0.36 | 3.5 | 3.8 | (Yagi et al., 2014) |
| Casuarina | *Casuarina equisetifolia* | 0.303 | 32.9 | 0.288 | 23.21 | 0.270 | 9.84 | 8.1 | (Ye et al., 2019) |
| Pumpkin | *Cucurbita pepo* | 0.263 | 32.8 | 0.93 | 16.8 | 0.47 | 12.4 | 4.4 | (Montero‐Pau et al., 2018) |
| European pear | *Pyrus communis* | 0.600 | 32.5 | 0.194 | 21. 2 | 0.127 | 14.1 | 7.1 | (Chagné et al., 2014) |
| Lotus | *Lotus japonicus* | 0.472 | 30.8 | 0.32 | 10.4 | 0.3 | 4.5 | 4.6 | (Holligan et al., 2006) |
| Medicago | *Medicago truncatula* | 0.465 | 30.5 | 0.80 | 26.5 | 0.69 | 5.7 | 4.1 | (Young et al., 2011) |
| Mangrove | *Rhizophora apiculate* | 0.274 | 29.69 | 0.121 | 18.02 | 0.44 | 12.01 | 6.01 | (Xu et al., 2017c) |
| Danshen | *Salvia miltiorrhiza* | 0.611 | 29.38 | 0.179 | 13.04 | 0.79 | 11.02 | 2.02 | (He et al., 2018) |
| False flax | *Camelina sativa* | 0.750 | 28.08 | 0.424 | 13.75 | 0.126 | 10.20 | 3.55 | (Kagale et al., 2014) |
| Drummond's avens | *Dryas drummondii* | 0.253 | 27.96 | 0.20 | 13.29 | 0.9 | 2.08 | 0.86 | (Griesmann et al., 2018) |
| Sandalwood | *Santalum album* | 0.221 | 27.46 | 0.60 | 4.49 | 0.9 | 3.25 | 1.24 | (Mahesh et al., 2018) |
| Dandelion | *Taraxacum officinale* | 0.865 | 27.35-38.5 | 0.236-0.333 | 18.74-28.98 | 0.162-250 | 11.56 | 9.72 | (de Carvalho et al., 2016) |
| Radish | *Raphanus sativus* | 0.402 | 26.6 | 0.107 | 4.1 | 0.16 | 1.9 | 2.0 | (Kitashiba et al., 2014) |
| Scaly oak | *Casuarina glauca* | 0.314 | 25.44 | 0.30 | 15.56 | 0.18 | 1.33 | 1.71 | (Griesmann et al., 2018) |
| Pennycress | *Thlaspi arvense* | 0.539 | 24.38 | 0.91 | 20.94 | 0.78 | 15.8 | 5.14 | (Dorn et al., 2015) |
| Sensitive plant | *Mimosa pudica* | 0.896 | 23.59 | 0.48 | 9.84 | 0.20 | 1.29 | 1.9 | (Griesmann et al., 2018) |
| Flax | *Linum usitatissimum* | 0.304 | 23.06 | 0.73 | 17.19 | 0.55 | 7.89 | 9.30 | (González and Deyholos, 2012) |
| Fig | *Ficus carica* | 0.356 | 20.93 | 0.51 | 1.15 | 0.2 | 0.59 | 0.54 | (Mori et al., 2017) |
| *-* | *Rhazya stricta* | 0.274 | 20.5 | 0.55 | 11.15 | 0.23 | 6.9 | 5.6 | (Sabir et al., 2016) |
| Sweet orange | *Citrus sinensis* | 0.367 | 20.5 | 0.61 | 18.21 | 0.54 | 9.77 | 7.84 | (Xu et al., 2013) |
| Woodland strawberry | *Fragaria vesca* | 0.240 | 20.74 | 0.46 | 14.66 | 0.32 | 5.99 | 4.58 | (Shulaev et al., 2011) |
| Melon | *Cucumis melo* | 0.375 | 19.7 | 0.88 | 14.7 | 0.62 | 7.2 | 5.5 | (Garcia-Mas et al., 2012) |
| Cork oak | *Quercus suber* | 0.953 | 8.53 | 0.81 | 7.59 | 0.72 | 3.4 | 2.67 | (Ramos et al., 2018) |
| Cowslip | *Primula veris* | 0.479 | 7 | 0.21 | 6.77 | 0.21 | 4.33 | 2.44 | (Nowak et al., 2015) |
| Carnivorous bladderwort | *Utricularia gibba* | 0.82 | 3.12 | 0.2 | 2.0.64 | 0.1 | 1.4 | 0.6 | (Ibarra-Laclette et al., 2013) |
| Horseweed | *Conyza canadensi* | 0.335 | 2.61 | 0.8 | 2.54 | 0.8 | 1.0 | 1.54 | (Peng et al., 2014) |
| Gymnosperm | | | | | | | | | |
| - | *Gnetum montanum* | 4.11 | 85.93 | 3.49 | 77.38 | 3.15 | 64.67 | 7.45 | (Wan et al., 2018) |
| Sugar pine | *Pinus lambertiana* | 31 | 79 | 24.4 | 35.8 | 11 | 27.3 | 8.5 | (Stevens et al., 2016) |
| Ginkgo | *Ginkgo biloba* | 10.61 | 75.3 | 7.99 | 60.65 | 6.43 | 45.6 | 12.7 | (Guan et al., 2016) |
| Douglas-fir | *Pseudotsuga menziesii* | 16 | 56.7 | 9.54 | 35.16 | 5.92 | 24.8 | 11.8 | (Neale et al., 2017) |
| Water fern | *Azolla filiculoides* | 0.753 | 53.6 | 0.239 | 47.0 | 0.209 | 25.01 | 22.0 | (Li et al., 2018) |
| Watermoss | *Salvinia cucullate* | 0.255 | 44.5 | 0.119 | 26.2 | 0.70 | 20.01 | 6.1 |  |
| **Iwa hiba** | *Selaginella tamariscina* | 0.301 | 60.58 | 0.181 | 25.47 | 0.76 | 23.31 | 1.88 | (Xu et al., 2018) |
| Hornwort | Anthoceros angustus | 0.107 | 60.52 | 0.72 | 45.09 | 0.53 | 34.04 | 11.05 | (Zhang et al., 2020a) |
| Earth moss | *Physcomitrella patens* | 0.480 | 50 | 0.252 | 47.5 | 0.240 | 43.1 | 4.4 | (Rensing et al., 2008) |
| Selaginella | *Selaginella moellendorffii* | 0.110 | 28.8 | 0.62 | 23.8 | 0. 49 | 21.10 | 2.7 | (Banks et al., 2011) |
| Liverwort | *Marchantia polymorph*a | 0.215 | 27 | 0.63 | 3.3 | 0.7 | 3.07 | 3.03 | (Montgomery et al., 2020) |
| Purple laver | *Porphyra umbilicalis* | 0.87.7 | 43.91 | 0.38 | 9.99 | 0.7 | 6.77 | 3.22 | (Brawley et al., 2017) |
| Seaweed | *Chondrus crispus* | 0.105 | 73 | 0.76 | 55.3 | 0.58 | 24.3 | 7.9 | (Collén et al., 2013) |

**References**

Alioto, T., Alexiou, K.G., Bardil, A., Barteri, F., Castanera, R., Cruz, F., et al. (2020). Transposons played a major role in the diversification between the closely related almond and peach genomes: results from the almond genome sequence. *The Plant Journal* 101(2)**,** 455-472.

Ambardar, S., Vakhlu, J., and Sowdhamini, R. (2021). De-novo draft genome sequence of Crocus Sativus L, Saffron, a golden condiment. *bioRxiv*.

Avni, R., Nave, M., Barad, O., Baruch, K., Twardziok, S.O., Gundlach, H., et al. (2017). Wild emmer genome architecture and diversity elucidate wheat evolution and domestication. *Science* 357(6346)**,** 93-97.

Baek, S., Choi, K., Kim, G.-B., Yu, H.-J., Cho, A., Jang, H., et al. (2018). Draft genome sequence of wild Prunus yedoensis reveals massive inter-specific hybridization between sympatric flowering cherries. *Genome biology* 19(1)**,** 1-17.

Banks, J.A., Nishiyama, T., Hasebe, M., Bowman, J.L., Gribskov, M., DePamphilis, C., et al. (2011). The Selaginella genome identifies genetic changes associated with the evolution of vascular plants. *science* 332(6032)**,** 960-963.

Bariah, I., Keidar-Friedman, D., and Kashkush, K. (2020). Where the wild things are: Transposable elements as drivers of structural and functional variations in the wheat genome. *Frontiers in Plant Science* 11**,** 1477.

Bauer, E., Schmutzer, T., Barilar, I., Mascher, M., Gundlach, H., Martis, M.M., et al. (2017). Towards a whole‐genome sequence for rye (Secale cereale L.). *The Plant Journal* 89(5)**,** 853-869.

Belser, C., Istace, B., Denis, E., Dubarry, M., Baurens, F.-C., Falentin, C., et al. (2018). Chromosome-scale assemblies of plant genomes using nanopore long reads and optical maps. *Nature plants* 4(11)**,** 879-887.

Bertioli, D.J., Cannon, S.B., Froenicke, L., Huang, G., Farmer, A.D., Cannon, E.K., et al. (2016). The genome sequences of Arachis duranensis and Arachis ipaensis, the diploid ancestors of cultivated peanut. *Nature genetics* 48(4)**,** 438-446.

Bertioli, D.J., Jenkins, J., Clevenger, J., Dudchenko, O., Gao, D., Seijo, G., et al. (2019). The genome sequence of segmental allotetraploid peanut Arachis hypogaea. *Nature genetics* 51(5)**,** 877-884.

Bolger, A., Scossa, F., Bolger, M.E., Lanz, C., Maumus, F., Tohge, T., et al. (2014). The genome of the stress-tolerant wild tomato species Solanum pennellii. *Nature genetics* 46(9)**,** 1034-1038.

Bombarely, A., Moser, M., Amrad, A., Bao, M., Bapaume, L., Barry, C.S., et al. (2016). Insight into the evolution of the Solanaceae from the parental genomes of Petunia hybrida. *Nature plants* 2(6)**,** 1-9.

Brawley, S.H., Blouin, N.A., Ficko-Blean, E., Wheeler, G.L., Lohr, M., Goodson, H.V., et al. (2017). Insights into the red algae and eukaryotic evolution from the genome of Porphyra umbilicalis (Bangiophyceae, Rhodophyta). *Proceedings of the National Academy of Sciences* 114(31)**,** E6361-E6370.

Bredeson, J.V., Lyons, J.B., Prochnik, S.E., Wu, G.A., Ha, C.M., Edsinger-Gonzales, E., et al. (2016). Sequencing wild and cultivated cassava and related species reveals extensive interspecific hybridization and genetic diversity. *Nature biotechnology* 34(5)**,** 562-570.

Buti, M., Moretto, M., Barghini, E., Mascagni, F., Natali, L., Brilli, M., et al. (2018). The genome sequence and transcriptome of Potentilla micrantha and their comparison to Fragaria vesca (the woodland strawberry). *GigaScience* 7(4)**,** giy010.

Byrne, S.L., Nagy, I., Pfeifer, M., Armstead, I., Swain, S., Studer, B., et al. (2015). A synteny‐based draft genome sequence of the forage grass Lolium perenne. *The Plant Journal* 84(4)**,** 816-826.

Cai, J., Liu, X., Vanneste, K., Proost, S., Tsai, W.-C., Liu, K.-W., et al. (2015). The genome sequence of the orchid Phalaenopsis equestris. *Nature genetics* 47(1)**,** 65-72.

Chagné, D., Crowhurst, R.N., Pindo, M., Thrimawithana, A., Deng, C., Ireland, H., et al. (2014). The draft genome sequence of European pear (Pyrus communis L.‘Bartlett’). *PloS one* 9(4)**,** e92644.

Chan, A.P., Crabtree, J., Zhao, Q., Lorenzi, H., Orvis, J., Puiu, D., et al. (2010). Draft genome sequence of the oilseed species Ricinus communis. *Nature biotechnology* 28(9)**,** 951-956.

Chang, Y., Liu, H., Liu, M., Liao, X., Sahu, S.K., Fu, Y., et al. (2019). The draft genomes of five agriculturally important African orphan crops. *GigaScience* 8(3)**,** giy152.

Chao, Y.T., Chen, W.C., Chen, C.Y., Ho, H.Y., Yeh, C.H., Kuo, Y.T., et al. (2018). Chromosome‐level assembly, genetic and physical mapping of Phalaenopsis aphrodite genome provides new insights into species adaptation and resources for orchid breeding. *Plant biotechnology journal* 16(12)**,** 2027-2041.

Chaw, S.-M., Liu, Y.-C., Wu, Y.-W., Wang, H.-Y., Lin, C.-Y.I., Wu, C.-S., et al. (2019). Stout camphor tree genome fills gaps in understanding of flowering plant genome evolution. *Nature plants* 5(1)**,** 63-73.

Chen, C.-H., Kuo, T.C.-Y., Yang, M.-H., Chien, T.-Y., Chu, M.-J., Huang, L.-C., et al. (2014). Identification of cucurbitacins and assembly of a draft genome for Aquilaria agallocha. *BMC genomics* 15(1)**,** 1-11.

Chen, G., Mostafa, S., Lu, Z., Du, R., Cui, J., Wang, Y., et al. (2020). The jasmine (Jasminum sambac) genome and flower fragrances. *bioRxiv*.

Chen, J., Hao, Z., Guang, X., Zhao, C., Wang, P., Xue, L., et al. (2019). Liriodendron genome sheds light on angiosperm phylogeny and species–pair differentiation. *Nature plants* 5(1)**,** 18-25.

Chen, J., Huang, Q., Gao, D., Wang, J., Lang, Y., Liu, T., et al. (2013). Whole-genome sequencing of Oryza brachyantha reveals mechanisms underlying Oryza genome evolution. *Nature Communications* 4(1)**,** 1-9.

Cheng, H., Sun, G., He, S., Gong, W., Peng, Z., Wang, R., et al. (2019). Comparative effect of allopolyploidy on transposable element composition and gene expression between Gossypium hirsutum and its two diploid progenitors. *Journal of integrative plant biology* 61(1)**,** 45-59.

Cheng, S.-P., Jia, K.-H., Liu, H., Zhang, R.-G., Li, Z.-C., Zhou, S.-S., et al. (2021). Haplotype-resolved genome assembly and allele-specific gene expression in cultivated ginger. *Horticulture Research* 8(1)**,** 1-15.

Cheng, S., van den Bergh, E., Zeng, P., Zhong, X., Xu, J., Liu, X., et al. (2013). The Tarenaya hassleriana genome provides insight into reproductive trait and genome evolution of crucifers. *The Plant Cell* 25(8)**,** 2813-2830.

Clouse, J., Adhikary, D., Page, J., Ramaraj, T., Deyholos, M., Udall, J., et al. (2016). The amaranth genome: genome, transcriptome, and physical map assembly. *The Plant Genome* 9(1)**,** plantgenome2015.2007.0062.

Cocker, J.M., Wright, J., Li, J., Swarbreck, D., Dyer, S., Caccamo, M., et al. (2018). Primula vulgaris (primrose) genome assembly, annotation and gene expression, with comparative genomics on the heterostyly supergene. *Scientific reports* 8(1)**,** 1-13.

Collén, J., Porcel, B., Carré, W., Ball, S.G., Chaparro, C., Tonon, T., et al. (2013). Genome structure and metabolic features in the red seaweed Chondrus crispus shed light on evolution of the Archaeplastida. *Proceedings of the National Academy of Sciences* 110(13)**,** 5247-5252.

Copetti, D., Búrquez, A., Bustamante, E., Charboneau, J.L., Childs, K.L., Eguiarte, L.E., et al. (2017). Extensive gene tree discordance and hemiplasy shaped the genomes of North American columnar cacti. *Proceedings of the National Academy of Sciences* 114(45)**,** 12003-12008.

Davey, M.W., Gudimella, R., Harikrishna, J.A., Sin, L.W., Khalid, N., and Keulemans, J. (2013). A draft Musa balbisiana genome sequence for molecular genetics in polyploid, inter-and intra-specific Musa hybrids. *BMC genomics* 14(1)**,** 1-20.

de Assis, R., Baba, V.Y., Cintra, L.A., Gonçalves, L.S.A., Rodrigues, R., and Vanzela, A.L.L. (2020). Genome relationships and LTR-retrotransposon diversity in three cultivated Capsicum L.(Solanaceae) species. *BMC genomics* 21(1)**,** 1-14.

de Carvalho, J.F., de Jager, V., van Gurp, T.P., Wagemaker, N.C., and Verhoeven, K.J. (2016). Recent and dynamic transposable elements contribute to genomic divergence under asexuality. *BMC genomics* 17(1)**,** 1-12.

De Vega, J.J., Ayling, S., Hegarty, M., Kudrna, D., Goicoechea, J.L., Ergon, Å., et al. (2015). Red clover (Trifolium pratense L.) draft genome provides a platform for trait improvement. *Scientific reports* 5(1)**,** 1-10.

Denoeud, F., Carretero-Paulet, L., Dereeper, A., Droc, G., Guyot, R., Pietrella, M., et al. (2014). The coffee genome provides insight into the convergent evolution of caffeine biosynthesis. *science* 345(6201)**,** 1181-1184.

Diaz-Garcia, L., Garcia-Ortega, L.F., González-Rodríguez, M., Delaye, L., Iorizzo, M., and Zalapa, J. (2021). Chromosome-level genome assembly of the American cranberry (Vaccinium macrocarpon Ait.) and its wild relative Vaccinium microcarpum. *Frontiers in Plant Science* 12**,** 137.

Domínguez, M., Dugas, E., Benchouaia, M., Leduque, B., Jiménez-Gómez, J.M., Colot, V., et al. (2020). The impact of transposable elements on tomato diversity. *Nature communications* 11(1)**,** 1-11.

Dong, A.-X., Xin, H.-B., Li, Z.-J., Liu, H., Sun, Y.-Q., Nie, S., et al. (2018). High-quality assembly of the reference genome for scarlet sage, Salvia splendens, an economically important ornamental plant. *GigaScience* 7(7)**,** giy068.

Dorn, K.M., Fankhauser, J.D., Wyse, D.L., and Marks, M.D. (2015). A draft genome of field pennycress (Thlaspi arvense) provides tools for the domestication of a new winter biofuel crop. *DNA Research* 22(2)**,** 121-131.

Edger, P.P., Poorten, T.J., VanBuren, R., Hardigan, M.A., Colle, M., McKain, M.R., et al. (2019). Origin and evolution of the octoploid strawberry genome. *Nature genetics* 51(3)**,** 541-547.

Faisal, N. (2019). Genomic and evolutionary diversity of LTR retrotransposons in date palm (Phoenix dactylifera). *Pakistan Journal of Botany* 51(5)**,** 1637-1644.

Feng, C., Feng, C., Lin, X., Liu, S., Li, Y., and Kang, M. (2021). A chromosome‐level genome assembly provides insights into ascorbic acid accumulation and fruit softening in guava (Psidium guajava). *Plant biotechnology journal* 19(4)**,** 717-730.

Finkers, R., van Kaauwen, M.P., Ament, K., Burger-Meijer, K., Egging, R.J., Huits, H., et al. (2021). Insights from the first genome assembly of Onion (Allium cepa). *bioRxiv*.

Fu, Y., Li, L., Hao, S., Guan, R., Fan, G., Shi, C., et al. (2017). Draft genome sequence of the Tibetan medicinal herb Rhodiola crenulata. *Gigascience* 6(6)**,** gix033.

Fukushima, K., Fang, X., Alvarez-Ponce, D., Cai, H., Carretero-Paulet, L., Chen, C., et al. (2017). Genome of the pitcher plant Cephalotus reveals genetic changes associated with carnivory. *Nature Ecology & Evolution* 1(3)**,** 1-9.

Gao, S., Wang, B., Xie, S., Xu, X., Zhang, J., Pei, L., et al. (2020). A high-quality reference genome of wild Cannabis sativa. *Horticulture research* 7(1)**,** 1-11.

Gao, Y., Wang, H., Liu, C., Chu, H., Dai, D., Song, S., et al. (2018). De novo genome assembly of the red silk cotton tree (Bombax ceiba). *GigaScience* 7(5)**,** giy051.

Garcia-Mas, J., Benjak, A., Sanseverino, W., Bourgeois, M., Mir, G., González, V.M., et al. (2012). The genome of melon (Cucumis melo L.). *Proceedings of the National Academy of Sciences* 109(29)**,** 11872-11877.

Garsmeur, O., Droc, G., Antonise, R., Grimwood, J., Potier, B., Aitken, K., et al. (2018). A mosaic monoploid reference sequence for the highly complex genome of sugarcane. *Nature communications* 9(1)**,** 1-10.

Gebre, Y.G., Bertolini, E., Pè, M.E., and Zuccolo, A. (2016). Identification and characterization of abundant repetitive sequences in Eragrostis tef cv. Enatite genome. *BMC plant biology* 16(1)**,** 1-13.

González, L.G., and Deyholos, M.K. (2012). Identification, characterization and distribution of transposable elements in the flax (Linum usitatissimum L.) genome. *BMC genomics* 13(1)**,** 1-17.

Griesmann, M., Chang, Y., Liu, X., Song, Y., Haberer, G., Crook, M.B., et al. (2018). Phylogenomics reveals multiple losses of nitrogen-fixing root nodule symbiosis. *Science* 361(6398).

Guan, R., Zhao, Y., Zhang, H., Fan, G., Liu, X., Zhou, W., et al. (2016). Draft genome of the living fossil Ginkgo biloba. *Gigascience* 5(1)**,** s13742-13016-10154-13741.

Guo, L., Qiu, J., Han, Z., Ye, Z., Chen, C., Liu, C., et al. (2015). A host plant genome (Zizania latifolia) after a century‐long endophyte infection. *The Plant Journal* 83(4)**,** 600-609.

Guo, L., Qiu, J., Ye, C., Jin, G., Mao, L., Zhang, H., et al. (2017). Echinochloa crus-galli genome analysis provides insight into its adaptation and invasiveness as a weed. *Nature communications* 8(1)**,** 1-10.

Guo, S., Zhao, S., Sun, H., Wang, X., Wu, S., Lin, T., et al. (2019). Resequencing of 414 cultivated and wild watermelon accessions identifies selection for fruit quality traits. *Nature genetics* 51(11)**,** 1616-1623.

Guo, X., Hu, Q., Hao, G., Wang, X., Zhang, D., Ma, T., et al. (2018). The genomes of two Eutrema species provide insight into plant adaptation to high altitudes. *DNA Research* 25(3)**,** 307-315.

Hane, J.K., Ming, Y., Kamphuis, L.G., Nelson, M.N., Garg, G., Atkins, C.A., et al. (2017). A comprehensive draft genome sequence for lupin (Lupinus angustifolius), an emerging health food: insights into plant–microbe interactions and legume evolution. *Plant biotechnology journal* 15(3)**,** 318-330.

Haudry, A., Platts, A.E., Vello, E., Hoen, D.R., Leclercq, M., Williamson, R.J., et al. (2013). An atlas of over 90,000 conserved noncoding sequences provides insight into crucifer regulatory regions. *Nature genetics* 45(8)**,** 891-898.

He, N., Zhang, C., Qi, X., Zhao, S., Tao, Y., Yang, G., et al. (2013). Draft genome sequence of the mulberry tree Morus notabilis. *Nature communications* 4(1)**,** 1-9.

He, Y., Peng, F., Deng, C., Xiong, L., Huang, Z.-y., Zhang, R.-q., et al. (2018). Building an octaploid genome and transcriptome of the medicinal plant Pogostemon cablin from Lamiales. *Scientific data* 5(1)**,** 1-11.

Hirakawa, H., Kaur, P., Shirasawa, K., Nichols, P., Nagano, S., Appels, R., et al. (2016). Draft genome sequence of subterranean clover, a reference for genus Trifolium. *Scientific Reports* 6(1)**,** 1-9.

Hirakawa, H., Shirasawa, K., Miyatake, K., Nunome, T., Negoro, S., Ohyama, A., et al. (2014). Draft genome sequence of eggplant (Solanum melongena L.): the representative solanum species indigenous to the old world. *DNA research* 21(6)**,** 649-660.

Hittalmani, S., Mahesh, H., Shirke, M.D., Biradar, H., Uday, G., Aruna, Y., et al. (2017). Genome and transcriptome sequence of finger millet (Eleusine coracana (L.) Gaertn.) provides insights into drought tolerance and nutraceutical properties. *BMC genomics* 18(1)**,** 1-16.

Holligan, D., Zhang, X., Jiang, N., Pritham, E.J., and Wessler, S.R. (2006). The transposable element landscape of the model legume Lotus japonicus. *Genetics* 174(4)**,** 2215-2228.

Huang, J., Zhang, C., Zhao, X., Fei, Z., Wan, K., Zhang, Z., et al. (2016). The jujube genome provides insights into genome evolution and the domestication of sweetness/acidity taste in fruit trees. *PLoS genetics* 12(12)**,** e1006433.

Hufnagel, B., Marques, A., Soriano, A., Marquès, L., Divol, F., Doumas, P., et al. (2020). High-quality genome sequence of white lupin provides insight into soil exploration and seed quality. *Nature communications* 11(1)**,** 1-12.

Ibarra-Laclette, E., Lyons, E., Hernández-Guzmán, G., Pérez-Torres, C.A., Carretero-Paulet, L., Chang, T.-H., et al. (2013). Architecture and evolution of a minute plant genome. *Nature* 498(7452)**,** 94-98.

Initiative, I.B. (2010). Genome sequencing and analysis of the model grass Brachypodium distachyon. *Nature (London)* 463(7282)**,** 763-768.

Iorizzo, M., Ellison, S., Senalik, D., Zeng, P., Satapoomin, P., Huang, J., et al. (2016). A high-quality carrot genome assembly provides new insights into carotenoid accumulation and asterid genome evolution. *Nature genetics* 48(6)**,** 657-666.

Islam, M.S., Saito, J.A., Emdad, E.M., Ahmed, B., Islam, M.M., Halim, A., et al. (2017). Comparative genomics of two jute species and insight into fibre biogenesis. *Nature plants* 3(2)**,** 1-7.

Jaiswal, S.K., Mahajan, S., Chakraborty, A., Kumar, S., and Sharma, V.K. (2021). The genome sequence of Aloe vera reveals adaptive evolution of drought tolerance mechanisms. *Iscience* 24(2)**,** 102079.

Jia, H.M., Jia, H.J., Cai, Q.L., Wang, Y., Zhao, H.B., Yang, W.F., et al. (2019). The red bayberry genome and genetic basis of sex determination. *Plant biotechnology journal* 17(2)**,** 397-409.

Jian-ye, C., Fang-fang, X., Yan-ze, C., Can-bin, C., Wang-jin, L., Xiao-di, H., et al. (2021). A chromosome-scale genome sequence of pitaya (Hylocereus undatus) provides novel insights into the genome evolution and regulation of betalain biosynthesis. *Horticulture Research* 8(1).

Kagale, S., Koh, C., Nixon, J., Bollina, V., Clarke, W.E., Tuteja, R., et al. (2014). The emerging biofuel crop Camelina sativa retains a highly undifferentiated hexaploid genome structure. *Nature communications* 5(1)**,** 1-11.

Kang, Y.J., Kim, S.K., Kim, M.Y., Lestari, P., Kim, K.H., Ha, B.-K., et al. (2014). Genome sequence of mungbean and insights into evolution within Vigna species. *Nature communications* 5(1)**,** 1-9.

Kang, Y.J., Satyawan, D., Shim, S., Lee, T., Lee, J., Hwang, W.J., et al. (2015). Draft genome sequence of adzuki bean, Vigna angularis. *Scientific reports* 5(1)**,** 1-8.

Kim, Y.-M., Kim, S., Koo, N., Shin, A.-Y., Yeom, S.-I., Seo, E., et al. (2017). Genome analysis of Hibiscus syriacus provides insights of polyploidization and indeterminate flowering in woody plants. *Dna Research* 24(1)**,** 71-80.

Kitashiba, H., Li, F., Hirakawa, H., Kawanabe, T., Zou, Z., Hasegawa, Y., et al. (2014). Draft sequences of the radish (Raphanus sativus L.) genome. *DNA research* 21(5)**,** 481-490.

Kreplak, J., Madoui, M.-A., Cápal, P., Novák, P., Labadie, K., Aubert, G., et al. (2019). A reference genome for pea provides insight into legume genome evolution. *Nature Genetics* 51(9)**,** 1411-1422.

Langridge, P., Shi, B., and Fincher, G. (2012). A physical, genetic and functional sequence assembly of the barley genome.

Lee, H., Golicz, A.A., Bayer, P.E., Jiao, Y., Tang, H., Paterson, A.H., et al. (2016). The genome of a southern hemisphere seagrass species (Zostera muelleri). *Plant physiology* 172(1)**,** 272-283.

Leisner, C.P., Hamilton, J.P., Crisovan, E., Manrique‐Carpintero, N.C., Marand, A.P., Newton, L., et al. (2018). Genome sequence of M6, a diploid inbred clone of the high‐glycoalkaloid‐producing tuber‐bearing potato species Solanum chacoense, reveals residual heterozygosity. *The Plant Journal* 94(3)**,** 562-570.

Li, D., Qian, J., Li, W., Jiang, Y., Gan, G., Li, W., et al. (2019a). Genome sequence and analysis of the eggplant (Solanum melongena L.). *bioRxiv***,** 824540.

Li, F.-W., Brouwer, P., Carretero-Paulet, L., Cheng, S., De Vries, J., Delaux, P.-M., et al. (2018). Fern genomes elucidate land plant evolution and cyanobacterial symbioses. *Nature plants* 4(7)**,** 460-472.

Li, M., Yang, Y., Xu, R., Mu, W., Li, Y., Mao, X., et al. (2021a). A chromosome‐level genome assembly for the tertiary relict plant Tetracentron sinense oliv.(trochodendraceae). *Molecular Ecology Resources* 21(4)**,** 1186-1199.

Li, Q., Li, H., Huang, W., Xu, Y., Zhou, Q., Wang, S., et al. (2019b). A chromosome-scale genome assembly of cucumber (Cucumis sativus L.). *GigaScience* 8(6)**,** giz072.

Li, S.-F., Gao, W.-J., Zhao, X.-P., Dong, T.-Y., Deng, C.-L., and Lu, L.-D. (2014). Analysis of transposable elements in the genome of Asparagus officinalis from high coverage sequence data. *PLoS One* 9(5)**,** e97189.

Li, W., Zhang, Q.J., Zhu, T., Tong, Y., Li, K., Shi, C., et al. (2020). Draft genomes of two outcrossing wild rice, Oryza rufipogon and O. longistaminata, reveal genomic features associated with mating‐system evolution. *Plant direct* 4(6)**,** e00232.

Li, Y., Sun, P., Lu, Z., Chen, J., Wang, Z., Du, X., et al. (2021b). The Corylus mandshurica genome provides insights into the evolution of Betulaceae genomes and hazelnut breeding. *Horticulture research* 8(1)**,** 1-13.

Lin, T., Xu, X., Ruan, J., Liu, S., Wu, S., Shao, X., et al. (2018). Genome analysis of Taraxacum kok-saghyz Rodin provides new insights into rubber biosynthesis. *National Science Review* 5(1)**,** 78-87.

Lin, Y., Min, J., Lai, R., Wu, Z., Chen, Y., Yu, L., et al. (2017). Genome-wide sequencing of longan (Dimocarpus longan Lour.) provides insights into molecular basis of its polyphenol-rich characteristics. *Gigascience* 6(5)**,** gix023.

Ling, H.-Q., Ma, B., Shi, X., Liu, H., Dong, L., Sun, H., et al. (2018). Genome sequence of the progenitor of wheat A subgenome Triticum urartu. *Nature* 557(7705)**,** 424-428.

Liu, C., Feng, C., Peng, W., Hao, J., Wang, J., Pan, J., et al. (2020a). Chromosome-level draft genome of a diploid plum (Prunus salicina). *GigaScience* 9(12)**,** giaa130.

Liu, J., Shi, C., Shi, C.-C., Li, W., Zhang, Q.-J., Zhang, Y., et al. (2020b). The chromosome-based rubber tree genome provides new insights into spurge genome evolution and rubber biosynthesis. *Molecular plant* 13(2)**,** 336-350.

Liu, X., Liu, Y., Huang, P., Ma, Y., Qing, Z., Tang, Q., et al. (2017). The genome of medicinal plant Macleaya cordata provides new insights into benzylisoquinoline alkaloids metabolism. *Molecular plant* 10(7)**,** 975-989.

Liu, X., Zhao, B., Zheng, H.-J., Hu, Y., Lu, G., Yang, C.-Q., et al. (2015). Gossypium barbadense genome sequence provides insight into the evolution of extra-long staple fiber and specialized metabolites. *Scientific reports* 5(1)**,** 1-14.

Luan, M.B., Jian, J.B., Chen, P., Chen, J.H., Chen, J.H., Gao, Q., et al. (2018). Draft genome sequence of ramie, Boehmeria nivea (L.) Gaudich. *Molecular ecology resources* 18(3)**,** 639-645.

Lv, S., Cheng, S., Wang, Z., Li, S., Jin, X., Lan, L., et al. (2020). Draft genome of the famous ornamental plant Paeonia suffruticosa. *Ecology and evolution* 10(11)**,** 4518-4530.

Ma, J., Wan, D., Duan, B., Bai, X., Bai, Q., Chen, N., et al. (2019). Genome sequence and genetic transformation of a widely distributed and cultivated poplar. *Plant biotechnology journal* 17(2)**,** 451-460.

Mahesh, H.B., Subba, P., Advani, J., Shirke, M.D., Loganathan, R.M., Chandana, S.L., et al. (2018). Multi-omics driven assembly and annotation of the sandalwood (Santalum album) genome. *Plant physiology* 176(4)**,** 2772-2788.

Martínez‐García, P.J., Crepeau, M.W., Puiu, D., Gonzalez‐Ibeas, D., Whalen, J., Stevens, K.A., et al. (2016). The walnut (Juglans regia) genome sequence reveals diversity in genes coding for the biosynthesis of non‐structural polyphenols. *The Plant Journal* 87(5)**,** 507-532.

Ming, R., VanBuren, R., Wai, C.M., Tang, H., Schatz, M.C., Bowers, J.E., et al. (2015). The pineapple genome and the evolution of CAM photosynthesis. *Nature genetics* 47(12)**,** 1435-1442.

Mochida, K., Sakurai, T., Seki, H., Yoshida, T., Takahagi, K., Sawai, S., et al. (2017). Draft genome assembly and annotation of Glycyrrhiza uralensis, a medicinal legume. *The Plant Journal* 89(2)**,** 181-194.

Montero‐Pau, J., Blanca, J., Bombarely, A., Ziarsolo, P., Esteras, C., Martí‐Gómez, C., et al. (2018). De novo assembly of the zucchini genome reveals a whole‐genome duplication associated with the origin of the Cucurbita genus. *Plant biotechnology journal* 16(6)**,** 1161-1171.

Montgomery, S.A., Tanizawa, Y., Galik, B., Wang, N., Ito, T., Mochizuki, T., et al. (2020). Chromatin organization in early land plants reveals an ancestral association between H3K27me3, transposons, and constitutive heterochromatin. *Current Biology* 30(4)**,** 573-588. e577.

Mori, K., Shirasawa, K., Nogata, H., Hirata, C., Tashiro, K., Habu, T., et al. (2017). Identification of RAN1 orthologue associated with sex determination through whole genome sequencing analysis in fig (Ficus carica L.). *Scientific reports* 7(1)**,** 1-15.

Motamayor, J.C., Mockaitis, K., Schmutz, J., Haiminen, N., Livingstone III, D., Cornejo, O., et al. (2013). The genome sequence of the most widely cultivated cacao type and its use to identify candidate genes regulating pod color. *Genome biology* 14(6)**,** 1-25.

Myburg, A.A., Grattapaglia, D., Tuskan, G.A., Hellsten, U., Hayes, R.D., Grimwood, J., et al. (2014). The genome of Eucalyptus grandis. *Nature* 510(7505)**,** 356-362.

Nakamura, N., Hirakawa, H., Sato, S., Otagaki, S., Matsumoto, S., Tabata, S., et al. (2018). Genome structure of Rosa multiflora, a wild ancestor of cultivated roses. *Dna Research* 25(2)**,** 113-121.

Natsume, S., Takagi, H., Shiraishi, A., Murata, J., Toyonaga, H., Patzak, J., et al. (2015). The draft genome of hop (Humulus lupulus), an essence for brewing. *Plant and cell physiology* 56(3)**,** 428-441.

Neale, D.B., McGuire, P.E., Wheeler, N.C., Stevens, K.A., Crepeau, M.W., Cardeno, C., et al. (2017). The Douglas-fir genome sequence reveals specialization of the photosynthetic apparatus in Pinaceae. *G3: Genes, Genomes, Genetics* 7(9)**,** 3157-3167.

Nowak, M.D., Russo, G., Schlapbach, R., Huu, C.N., Lenhard, M., and Conti, E. (2015). The draft genome of Primula veris yields insights into the molecular basis of heterostyly. *Genome biology* 16(1)**,** 1-17.

Nystedt, B., Street, N.R., Wetterbom, A., Zuccolo, A., Lin, Y.-C., Scofield, D.G., et al. (2013). The Norway spruce genome sequence and conifer genome evolution. *Nature* 497(7451)**,** 579-584.

Oliver, K.R., McComb, J.A., and Greene, W.K. (2013). Transposable elements: powerful contributors to angiosperm evolution and diversity. *Genome biology and evolution* 5(10)**,** 1886-1901.

Paterson, A.H., Bowers, J.E., Bruggmann, R., Dubchak, I., Grimwood, J., Gundlach, H., et al. (2009). The Sorghum bicolor genome and the diversification of grasses. *Nature* 457(7229)**,** 551-556.

Pei, L., Wang, B., Ye, J., Hu, X., Fu, L., Li, K., et al. (2021). Genome and transcriptome of Papaver somniferum Chinese landrace CHM indicates that massive genome expansion contributes to high benzylisoquinoline alkaloid biosynthesis. *Horticulture Research* 8(1)**,** 1-13.

Peng, Y., Lai, Z., Lane, T., Nageswara-Rao, M., Okada, M., Jasieniuk, M., et al. (2014). De novo genome assembly of the economically important weed horseweed using integrated data from multiple sequencing platforms. *Plant Physiology* 166(3)**,** 1241-1254.

Peng, Z., Lu, Y., Li, L., Zhao, Q., Feng, Q., Gao, Z., et al. (2013). The draft genome of the fast-growing non-timber forest species moso bamboo (Phyllostachys heterocycla). *Nature genetics* 45(4)**,** 456-461.

Pereira, V. (2004). Insertion bias and purifying selection of retrotransposons in the Arabidopsis thaliana genome. *Genome biology* 5(10)**,** 1-10.

Plomion, C., Aury, J.-M., Amselem, J., Leroy, T., Murat, F., Duplessis, S., et al. (2018). Oak genome reveals facets of long lifespan. *Nature Plants* 4(7)**,** 440-452.

Pootakham, W., Naktang, C., Kongkachana, W., Sonthirod, C., Yoocha, T., Sangsrakru, D., et al. (2021). De novo chromosome-level assembly of the Centella asiatica genome. *Genomics* 113(4)**,** 2221-2228.

Rahman, A.Y.A., Usharraj, A.O., Misra, B.B., Thottathil, G.P., Jayasekaran, K., Feng, Y., et al. (2013). Draft genome sequence of the rubber tree Hevea brasiliensis. *BMC genomics* 14(1)**,** 1-15.

Ramos, A.M., Usié, A., Barbosa, P., Barros, P.M., Capote, T., Chaves, I., et al. (2018). The draft genome sequence of cork oak. *Scientific data* 5(1)**,** 1-12.

Rao, G., Zhang, J., Liu, X., Lin, C., Xin, H., Xue, L., et al. (2021). De novo assembly of a new Olea europaea genome accession using nanopore sequencing. *Horticulture research* 8(1)**,** 1-12.

Razali, R., Bougouffa, S., Morton, M.J., Lightfoot, D.J., Alam, I., Essack, M., et al. (2018). The genome sequence of the wild tomato Solanum pimpinellifolium provides insights into salinity tolerance. *Frontiers in plant science* 9**,** 1402.

Rensing, S.A., Lang, D., Zimmer, A.D., Terry, A., Salamov, A., Shapiro, H., et al. (2008). The Physcomitrella genome reveals evolutionary insights into the conquest of land by plants. *Science* 319(5859)**,** 64-69.

Reyes-Chin-Wo, S., Wang, Z., Yang, X., Kozik, A., Arikit, S., Song, C., et al. (2017). Genome assembly with in vitro proximity ligation data and whole-genome triplication in lettuce. *Nature Communications* 8(1)**,** 1-11.

Sabir, J.S., Jansen, R.K., Arasappan, D., Calderon, V., Noutahi, E., Zheng, C., et al. (2016). The nuclear genome of Rhazya stricta and the evolution of alkaloid diversity in a medically relevant clade of Apocynaceae. *Scientific reports* 6(1)**,** 1-10.

Saint-Oyant, L.H., Ruttink, T., Hamama, L., Kirov, I., Lakhwani, D., Zhou, N.-N., et al. (2018). A high-quality genome sequence of Rosa chinensis to elucidate ornamental traits. *Nature plants* 4(7)**,** 473-484.

Scaglione, D., Reyes-Chin-Wo, S., Acquadro, A., Froenicke, L., Portis, E., Beitel, C., et al. (2016). The genome sequence of the outbreeding globe artichoke constructed de novo incorporating a phase-aware low-pass sequencing strategy of F 1 progeny. *Scientific Reports* 6(1)**,** 1-17.

Schmutz, J., Cannon, S.B., Schlueter, J., Ma, J., Mitros, T., Nelson, W., et al. (2010). Genome sequence of the palaeopolyploid soybean. *nature* 463(7278)**,** 178-183.

Schmutz, J., McClean, P.E., Mamidi, S., Wu, G.A., Cannon, S.B., Grimwood, J., et al. (2014). A reference genome for common bean and genome-wide analysis of dual domestications. *Nature genetics* 46(7)**,** 707-713.

Schnable, P.S., Ware, D., Fulton, R.S., Stein, J.C., Wei, F., Pasternak, S., et al. (2009). The B73 maize genome: complexity, diversity, and dynamics. *science* 326(5956)**,** 1112-1115.

Shen, Q., Zhang, L., Liao, Z., Wang, S., Yan, T., Shi, P., et al. (2018). The genome of Artemisia annua provides insight into the evolution of Asteraceae family and artemisinin biosynthesis. *Molecular plant* 11(6)**,** 776-788.

Shirasawa, K., Isuzugawa, K., Ikenaga, M., Saito, Y., Yamamoto, T., Hirakawa, H., et al. (2017). The genome sequence of sweet cherry (Prunus avium) for use in genomics-assisted breeding. *DNA Research* 24(5)**,** 499-508.

Shulaev, V., Sargent, D.J., Crowhurst, R.N., Mockler, T.C., Folkerts, O., Delcher, A.L., et al. (2011). The genome of woodland strawberry (Fragaria vesca). *Nature genetics* 43(2)**,** 109-116.

Sierro, N., Battey, J.N., Ouadi, S., Bakaher, N., Bovet, L., Willig, A., et al. (2014). The tobacco genome sequence and its comparison with those of tomato and potato. *Nature communications* 5(1)**,** 1-9.

Sierro, N., Battey, J.N., Ouadi, S., Bovet, L., Goepfert, S., Bakaher, N., et al. (2013). Reference genomes and transcriptomes of Nicotiana sylvestris and Nicotiana tomentosiformis. *Genome biology* 14(6)**,** 1-17.

Silva-Junior, O.B., Grattapaglia, D., Novaes, E., and Collevatti, R.G. (2018). Genome assembly of the pink ipê (Handroanthus impetiginosus, Bignoniaceae), a highly valued, ecologically keystone neotropical timber forest tree. *Gigascience* 7(1)**,** gix125.

Singh, R., Ong-Abdullah, M., Low, E.-T.L., Manaf, M.A.A., Rosli, R., Nookiah, R., et al. (2013). Oil palm genome sequence reveals divergence of interfertile species in Old and New worlds. *Nature* 500(7462)**,** 335-339.

Sollars, E.S., Harper, A.L., Kelly, L.J., Sambles, C.M., Ramirez-Gonzalez, R.H., Swarbreck, D., et al. (2017). Genome sequence and genetic diversity of European ash trees. *Nature* 541(7636)**,** 212-216.

Song, C., Liu, Y., Song, A., Dong, G., Zhao, H., Sun, W., et al. (2018). The Chrysanthemum nankingense genome provides insights into the evolution and diversification of chrysanthemum flowers and medicinal traits. *Molecular Plant* 11(12)**,** 1482-1491.

Song, J.-M., Guan, Z., Hu, J., Guo, C., Yang, Z., Wang, S., et al. (2020). Eight high-quality genomes reveal pan-genome architecture and ecotype differentiation of Brassica napus. *Nature Plants* 6(1)**,** 34-45.

Staton, S.E., Bakken, B.H., Blackman, B.K., Chapman, M.A., Kane, N.C., Tang, S., et al. (2012). The sunflower (Helianthus annuus L.) genome reflects a recent history of biased accumulation of transposable elements. *The plant journal* 72(1)**,** 142-153.

Stein, J.C., Yu, Y., Copetti, D., Zwickl, D.J., Zhang, L., Zhang, C., et al. (2018). Genomes of 13 domesticated and wild rice relatives highlight genetic conservation, turnover and innovation across the genus Oryza. *Nature genetics* 50(2)**,** 285-296.

Stevens, K.A., Wegrzyn, J.L., Zimin, A., Puiu, D., Crepeau, M., Cardeno, C., et al. (2016). Sequence of the sugar pine megagenome. *Genetics* 204(4)**,** 1613-1626.

Sun, D., Wang, C., Zhang, X., Zhang, W., Jiang, H., Yao, X., et al. (2019a). Draft genome sequence of cauliflower (Brassica oleracea L. var. botrytis) provides new insights into the C genome in Brassica species. *Horticulture research* 6(1)**,** 1-11.

Sun, G., Xu, Y., Liu, H., Sun, T., Zhang, J., Hettenhausen, C., et al. (2018). Large-scale gene losses underlie the genome evolution of parasitic plant Cuscuta australis. *Nature communications* 9(1)**,** 1-8.

Sun, W., Leng, L., Yin, Q., Xu, M., Huang, M., Xu, Z., et al. (2019b). The genome of the medicinal plant Andrographis paniculata provides insight into the biosynthesis of the bioactive diterpenoid neoandrographolide. *The Plant Journal* 97(5)**,** 841-857.

Sun, X., Zhu, S., Li, N., Cheng, Y., Zhao, J., Qiao, X., et al. (2020). A chromosome-level genome assembly of garlic (Allium sativum) provides insights into genome evolution and allicin biosynthesis. *Molecular Plant* 13(9)**,** 1328-1339.

Tamiru, M., Natsume, S., Takagi, H., White, B., Yaegashi, H., Shimizu, M., et al. (2017). Genome sequencing of the staple food crop white Guinea yam enables the development of a molecular marker for sex determination. *BMC biology* 15(1)**,** 1-20.

Tanaka, H., Hirakawa, H., Kosugi, S., Nakayama, S., Ono, A., Watanabe, A., et al. (2016). Sequencing and comparative analyses of the genomes of zoysiagrasses. *DNA Research* 23(2)**,** 171-180.

Teh, B.T., Lim, K., Yong, C.H., Ng, C.C.Y., Rao, S.R., Rajasegaran, V., et al. (2017). The draft genome of tropical fruit durian (Durio zibethinus). *Nature genetics* 49(11)**,** 1633-1641.

Tomato Genome Consortium, x. (2012). The tomato genome sequence provides insights into fleshy fruit evolution. *Nature* 485(7400)**,** 635.

Van Hoeck, A., Horemans, N., Monsieurs, P., Cao, H.X., Vandenhove, H., and Blust, R. (2015). The first draft genome of the aquatic model plant Lemna minor opens the route for future stress physiology research and biotechnological applications. *Biotechnology for biofuels* 8(1)**,** 1-13.

VanBuren, R., Bryant, D., Bushakra, J.M., Vining, K.J., Edger, P.P., Rowley, E.R., et al. (2016). The genome of black raspberry (Rubus occidentalis). *The Plant Journal* 87(6)**,** 535-547.

Varshney, R.K., Chen, W., Li, Y., Bharti, A.K., Saxena, R.K., Schlueter, J.A., et al. (2012). Draft genome sequence of pigeonpea (Cajanus cajan), an orphan legume crop of resource-poor farmers. *Nature biotechnology* 30(1)**,** 83.

Varshney, R.K., Shi, C., Thudi, M., Mariac, C., Wallace, J., Qi, P., et al. (2017). Pearl millet genome sequence provides a resource to improve agronomic traits in arid environments. *Nature biotechnology* 35(10)**,** 969-976.

Varshney, R.K., Song, C., Saxena, R.K., Azam, S., Yu, S., Sharpe, A.G., et al. (2013). Draft genome sequence of chickpea (Cicer arietinum) provides a resource for trait improvement. *Nature biotechnology* 31(3)**,** 240-246.

Verde, I., Abbott, A.G., Scalabrin, S., Jung, S., Shu, S., Marroni, F., et al. (2013). The high-quality draft genome of peach (Prunus persica) identifies unique patterns of genetic diversity, domestication and genome evolution. *Nature genetics* 45(5)**,** 487-494.

Vogel, A., Schwacke, R., Denton, A.K., Usadel, B., Hollmann, J., Fischer, K., et al. (2018). Footprints of parasitism in the genome of the parasitic flowering plant Cuscuta campestris. *Nature communications* 9(1)**,** 1-11.

Wan, T., Liu, Z.-M., Li, L.-F., Leitch, A.R., Leitch, I.J., Lohaus, R., et al. (2018). A genome for gnetophytes and early evolution of seed plants. *Nature Plants* 4(2)**,** 82-89.

Wang, L., He, F., Huang, Y., He, J., Yang, S., Zeng, J., et al. (2018). Genome of wild mandarin and domestication history of mandarin. *Molecular plant* 11(8)**,** 1024-1037.

Wang, P., Luo, Y., Huang, J., Gao, S., Zhu, G., Dang, Z., et al. (2020). The genome evolution and domestication of tropical fruit mango. *Genome biology* 21(1)**,** 1-17.

Wang, X., Wang, H., Wang, J., Sun, R., Wu, J., Liu, S., et al. (2011). The genome of the mesopolyploid crop species Brassica rapa. *Nature genetics* 43(10)**,** 1035-1039.

Wang, X., Xu, Y., Zhang, S., Cao, L., Huang, Y., Cheng, J., et al. (2017). Genomic analyses of primitive, wild and cultivated citrus provide insights into asexual reproduction. *Nature Genetics* 49(5)**,** 765-772.

Wicker, T., Gundlach, H., Spannagl, M., Uauy, C., Borrill, P., Ramírez-González, R.H., et al. (2018). Impact of transposable elements on genome structure and evolution in bread wheat. *Genome biology* 19(1)**,** 1-18.

Willing, E.-M., Rawat, V., Mandáková, T., Maumus, F., James, G.V., Nordström, K.J., et al. (2015). Genome expansion of Arabis alpina linked with retrotransposition and reduced symmetric DNA methylation. *Nature plants* 1(2)**,** 1-7.

Wu, G.A., Prochnik, S., Jenkins, J., Salse, J., Hellsten, U., Murat, F., et al. (2014). Sequencing of diverse mandarin, pummelo and orange genomes reveals complex history of admixture during citrus domestication. *Nature biotechnology* 32(7)**,** 656-662.

Wu, H., Ma, T., Kang, M., Ai, F., Zhang, J., Dong, G., et al. (2019a). A high-quality Actinidia chinensis (kiwifruit) genome. *Horticulture research* 6(1)**,** 1-9.

Wu, J., Wang, Z., Shi, Z., Zhang, S., Ming, R., Zhu, S., et al. (2013). The genome of the pear (Pyrus bretschneideri Rehd.). *Genome research* 23(2)**,** 396-408.

Wu, M., Kostyun, J.L., and Moyle, L.C. (2019b). Genome sequence of Jaltomata addresses rapid reproductive trait evolution and enhances comparative genomics in the hyper-diverse Solanaceae. *Genome biology and evolution* 11(2)**,** 335-349.

Wu, S., Lau, K.H., Cao, Q., Hamilton, J.P., Sun, H., Zhou, C., et al. (2018). Genome sequences of two diploid wild relatives of cultivated sweetpotato reveal targets for genetic improvement. *Nature communications* 9(1)**,** 1-12.

Wu, S., Shamimuzzaman, M., Sun, H., Salse, J., Sui, X., Wilder, A., et al. (2017). The bottle gourd genome provides insights into Cucurbitaceae evolution and facilitates mapping of a Papaya ring‐spot virus resistance locus. *The Plant Journal* 92(5)**,** 963-975.

Wu, W., Yang, Y.-L., He, W.-M., Rouard, M., Li, W.-M., Xu, M., et al. (2016). Whole genome sequencing of a banana wild relative Musa itinerans provides insights into lineage-specific diversification of the Musa genus. *Scientific Reports* 6(1)**,** 1-11.

Wuyun, T.-n., Wang, L., Liu, H., Wang, X., Zhang, L., Bennetzen, J.L., et al. (2018). The hardy rubber tree genome provides insights into the evolution of polyisoprene biosynthesis. *Molecular plant* 11(3)**,** 429-442.

Xia, M., Han, X., He, H., Yu, R., Zhen, G., Jia, X., et al. (2018). Improved de novo genome assembly and analysis of the Chinese cucurbit Siraitia grosvenorii, also known as monk fruit or luo-han-guo. *Gigascience* 7(6)**,** giy067.

Xiao, L., Yang, G., Zhang, L., Yang, X., Zhao, S., Ji, Z., et al. (2015). The resurrection genome of Boea hygrometrica: A blueprint for survival of dehydration. *Proceedings of the National Academy of Sciences* 112(18)**,** 5833-5837.

Xiao, Y., Xu, P., Fan, H., Baudouin, L., Xia, W., Bocs, S., et al. (2017). The genome draft of coconut (Cocos nucifera). *Gigascience* 6(11)**,** gix095.

Xu, C.-Q., Liu, H., Zhou, S.-S., Zhang, D.-X., Zhao, W., Wang, S., et al. (2019). Genome sequence of Malania oleifera, a tree with great value for nervonic acid production. *GigaScience* 8(2)**,** giy164.

Xu, C., Jiao, C., Sun, H., Cai, X., Wang, X., Ge, C., et al. (2017a). Draft genome of spinach and transcriptome diversity of 120 Spinacia accessions. *Nature Communications* 8(1)**,** 1-10.

Xu, Q., Chen, L.-L., Ruan, X., Chen, D., Zhu, A., Chen, C., et al. (2013). The draft genome of sweet orange (Citrus sinensis). *Nature genetics* 45(1)**,** 59-66.

Xu, S., Brockmöller, T., Navarro-Quezada, A., Kuhl, H., Gase, K., Ling, Z., et al. (2017b). Wild tobacco genomes reveal the evolution of nicotine biosynthesis. *Proceedings of the National Academy of Sciences* 114(23)**,** 6133-6138.

Xu, S., He, Z., Zhang, Z., Guo, Z., Guo, W., Lyu, H., et al. (2017c). The origin, diversification and adaptation of a major mangrove clade (Rhizophoreae) revealed by whole-genome sequencing. *National Science Review* 4(5)**,** 721-734.

Xu, Z., Xin, T., Bartels, D., Li, Y., Gu, W., Yao, H., et al. (2018). Genome analysis of the ancient tracheophyte Selaginella tamariscina reveals evolutionary features relevant to the acquisition of desiccation tolerance. *Molecular plant* 11(7)**,** 983-994.

Yagi, M., Kosugi, S., Hirakawa, H., Ohmiya, A., Tanase, K., Harada, T., et al. (2014). Sequence analysis of the genome of carnation (Dianthus caryophyllus L.). *DNA Research* 21(3)**,** 231-241.

Yamashiro, T., Shiraishi, A., Satake, H., and Nakayama, K. (2019). Draft genome of Tanacetum cinerariifolium, the natural source of mosquito coil. *Scientific reports* 9(1)**,** 1-17.

Yang, J., Moeinzadeh, M.-H., Kuhl, H., Helmuth, J., Xiao, P., Haas, S., et al. (2017a). Haplotype-resolved sweet potato genome traces back its hexaploidization history. *Nature plants* 3(9)**,** 696-703.

Yang, J., Zhang, G., Zhang, J., Liu, H., Chen, W., Wang, X., et al. (2017b). Hybrid de novo genome assembly of the Chinese herbal fleabane Erigeron breviscapus. *Gigascience* 6(6)**,** gix028.

Yang, Y., Bocs, S., Fan, H., Armero, A., Baudouin, L., Xu, P., et al. (2021). Coconut genome assembly enables evolutionary analysis of palms and highlights signaling pathways involved in salt tolerance. *Communications biology* 4(1)**,** 1-14.

Yasui, Y., Hirakawa, H., Ueno, M., Matsui, K., Katsube-Tanaka, T., Yang, S.J., et al. (2016). Assembly of the draft genome of buckwheat and its applications in identifying agronomically useful genes. *DNA Research* 23(3)**,** 215-224.

Ye, G., Zhang, H., Chen, B., Nie, S., Liu, H., Gao, W., et al. (2019). De novo genome assembly of the stress tolerant forest species Casuarina equisetifolia provides insight into secondary growth. *The Plant Journal* 97(4)**,** 779-794.

Yu, J., Hu, S., Wang, J., Wong, G.K.-S., Li, S., Liu, B., et al. (2002). A draft sequence of the rice genome (Oryza sativa L. ssp. indica). *science* 296(5565)**,** 79-92.

Yuan, Y., Jin, X., Liu, J., Zhao, X., Zhou, J., Wang, X., et al. (2018a). The Gastrodia elata genome provides insights into plant adaptation to heterotrophy. *Nature Communications* 9(1)**,** 1-11.

Yuan, Z., Fang, Y., Zhang, T., Fei, Z., Han, F., Liu, C., et al. (2018b). The pomegranate (Punica granatum L.) genome provides insights into fruit quality and ovule developmental biology. *Plant biotechnology journal* 16(7)**,** 1363-1374.

Zhang, G.-Q., Liu, K.-W., Li, Z., Lohaus, R., Hsiao, Y.-Y., Niu, S.-C., et al. (2017a). The Apostasia genome and the evolution of orchids. *Nature* 549(7672)**,** 379-383.

Zhang, G.-Q., Xu, Q., Bian, C., Tsai, W.-C., Yeh, C.-M., Liu, K.-W., et al. (2016). The Dendrobium catenatum Lindl. genome sequence provides insights into polysaccharide synthase, floral development and adaptive evolution. *Scientific reports* 6(1)**,** 1-10.

Zhang, J., Fu, X.-X., Li, R.-Q., Zhao, X., Liu, Y., Li, M.-H., et al. (2020a). The hornwort genome and early land plant evolution. *Nature plants* 6(2)**,** 107-118.

Zhang, L., Chen, F., Zhang, X., Li, Z., Zhao, Y., Lohaus, R., et al. (2020b). The water lily genome and the early evolution of flowering plants. *Nature* 577(7788)**,** 79-84.

Zhang, L., Hu, J., Han, X., Li, J., Gao, Y., Richards, C.M., et al. (2019). A high-quality apple genome assembly reveals the association of a retrotransposon and red fruit colour. *Nature communications* 10(1)**,** 1-13.

Zhang, L., Li, X., Ma, B., Gao, Q., Du, H., Han, Y., et al. (2017b). The tartary buckwheat genome provides insights into rutin biosynthesis and abiotic stress tolerance. *Molecular Plant* 10(9)**,** 1224-1237.

Zhang, L., Xu, P., Cai, Y., Ma, L., Li, S., Li, S., et al. (2017c). The draft genome assembly of Rhododendron delavayi Franch. var. delavayi. *GigaScience* 6(10)**,** gix076.

Zhang, Q.-J., and Gao, L.-Z. (2017). Rapid and recent evolution of LTR retrotransposons drives rice genome evolution during the speciation of AA-genome Oryza species. *G3: Genes, Genomes, Genetics* 7(6)**,** 1875-1885.

Zhang, Q.-J., Li, W., Li, K., Nan, H., Shi, C., Zhang, Y., et al. (2020c). The chromosome-level reference genome of tea tree unveils recent bursts of non-autonomous LTR retrotransposons in driving genome size evolution. *Molecular plant* 13(7)**,** 935-938.

Zhang, Q., Chen, W., Sun, L., Zhao, F., Huang, B., Yang, W., et al. (2012). The genome of Prunus mume. *Nature communications* 3(1)**,** 1-8.

Zhang, X., Liu, T., Wang, J., Wang, P., Qiu, Y., Zhao, W., et al. (2021). Pan-genome of Raphanus highlights genetic variation and introgression among domesticated, wild, and weedy radishes. *Molecular Plant*.

Zhao, G., Zou, C., Li, K., Wang, K., Li, T., Gao, L., et al. (2017). The Aegilops tauschii genome reveals multiple impacts of transposons. *Nature Plants* 3(12)**,** 946-955.

Zhao, H., Wang, S., Wang, J., Chen, C., Hao, S., Chen, L., et al. (2018). The chromosome-level genome assemblies of two rattans (Calamus simplicifolius and Daemonorops jenkinsiana). *GigaScience* 7(9)**,** giy097.

Zhou, M., Hu, B., and Zhu, Y. (2017). Genome-wide characterization and evolution analysis of long terminal repeat retroelements in moso bamboo (Phyllostachys edulis). *Tree Genetics & Genomes* 13(2)**,** 43.
